# Tropical biodiversity loss from land-use change is severely underestimated by local-scale assessments

**DOI:** 10.1038/s41559-025-02779-4

**Published:** 2025-07-22

**Authors:** Jacob B. Socolar, Simon C. Mills, James J. Gilroy, Diego E. Martínez-Revelo, Claudia A. Medina-Uribe, Edicson Parra-Sanchez, Marcela Ramirez-Gutierrez, Jørgen Sand Sæbø, Henry S. Meneses, Giovanny Pérez, Jos Barlow, Jose M. Ochoa Quintero, Robert P. Freckleton, Torbjørn Haugaasen, David P. Edwards

**Affiliations:** 1 Faculty of Environmental Sciences and Natural Resource Management, Norwegian University of Life Sciences, Ås, Norway; 2 BeZero Carbon Ltd, London, UK; 3 Ecology and Evolutionary Biology, School of Biosciences, University of Sheffield, Sheffield, UK; 4 Rainforest Builder, London, UK; 5 School of Environmental Sciences, University of East Anglia, Norwich, UK; 6 Grupo de Investigación en Ecología de Agroecosistemas y Hábitats Naturales (GEAHNA), Departamento de Biología, Facultad de Ciencias Naturales y Exactas, Universidad del Valle, Cali, Colombia; 7 Instituto de Investigación de Recursos Biológicos Alexander von Humboldt, Bogotá, Colombia; 8 Department of Plant Sciences and Centre for Global Wood Security, University of Cambridge, Cambridge, UK; 9 Universidad de los Llanos, Villavicencio, Colombia; 10 Parques Nacionales Naturales de Colombia, Bogotá, Colombia; 11 Lancaster Environment Centre, Lancaster University, Lancaster, UK; 12 Instituto de Biociencias, Universidad Federal de Mato Grosso do Sul, Campo Grande, Brazil; 13 Conservation Research Institute, University of Cambridge, Cambridge, UK

**Keywords:** Conservation biology, Tropical ecology, Community ecology

## Abstract

Human impacts on nature span vast spatial scales that transcend abiotic gradients and biogeographic barriers, yet estimates of biodiversity loss from land-use change overwhelmingly derive from local-scale studies. Using a field dataset of 971 bird species sampled in forest and cattle pasture across 13 biogeographic regions of Colombia, we quantify biodiversity losses from local to near-national scales. Losses are on average 60% worse at the pan-Colombian scale than in individual regions, with underestimation remaining until six to seven biogeographic regions are sampled. Regional losses greatly exceed local losses when beta-diversity is high due to reduced species turnover in pasture across geographic space and elevation. Extrapolation from local-scale studies causes major underestimation of biodiversity loss, emphasizing the need to incorporate spatial structure into measures of change.

## Abstract (web summary)

Analysis of data on 971 bird species in natural habitat and cattle pasture in Colombia finds that near-national-scale losses of bird diversity greatly exceed losses recorded at the local scale, suggesting that extrapolations from local studies will severely underestimate biodiversity losses.

## Main

The tropics are the most hyperdiverse region on the planet^1,2^, with tropical forests harbouring ~62% of terrestrial vertebrate species on just 18% of the land surface^3^. In addition to exceptionally high levels of local (alpha) diversity^4^, much of this tropical hyperdiversity is explained by a rapid turnover of species composition across space (beta-diversity), with markedly different communities found in different locations. Rapid species turnover in the tropics occurs across steep environmental gradients, including elevation and precipitation^5,6^, and across dispersal-limiting features, such as major river or mountain top barriers^5,7^. There are thus sharp turnovers of species between different but spatially proximate habitats, such as the terra firme, varzea, river island and white-sands forests of the western Amazon^8^.

Land-use change is a leading driver of the global biodiversity crisis^9^, particularly in the highly diverse tropics^10^. Land conversion erodes habitat complexity, reduces microclimate variability, and limits niche availability and dispersal potential across remaining natural habitat^11^. The result is that a characteristic suite of species tends to persist in converted habitats, no matter where they are located. Habitat conversion thus results in biotic homogenization, with increasing compositional similarity across spatially disparate communities^11–13^. However, efforts to quantify the severity of biodiversity change across land-use transitions are beset by problems of spatial scale^14–18^, and biotic homogenization can produce spatial scaling phenomena that decouple local from regional biodiversity change^14–17,19^. Spatial decoupling can occur via the increasing loss of small-ranged forest-dwelling species across broader relative to local spatial scales (subtractive homogenization), coupled with the colonization of farmland by edge-tolerant species that are large-ranged and become ubiquitous from local to broader scales (additive homogenization)^14^, generating patterns of more severe biotic homogenization at regional versus local scales.

Field-based biodiversity assessments across land-use transitions overwhelmingly focus on understanding local-scale biodiversity change rather than on assessing regional effect sizes^20,21^. In turn, global meta-analyses quantify biodiversity change by averaging across local-scale effects, without directly assessing regional impacts^22^. Aggregating local estimates of species richness change—the most common indicator of biodiversity loss—fails to reflect global trends with enough accuracy to be useful for policymaking^23^. As a result, this approach leaves considerable uncertainty about the severity of biodiversity change at larger spatial scales^14^.

Without direct measurements of regional-scale biodiversity change, empirical support for biotic homogenization in human-modified landscapes derives primarily from two approaches. First, community dissimilarity metrics often reveal pairwise homogenization of species assemblages across sites undergoing land-use change relative to control sites that remain in a natural state (that is, a decrease in mean pairwise dissimilarity)^15–18,24^. Second, indices accounting for the range size of species lost and gained reveal disproportionate declines in range-restricted species^11,25,26^, suggesting that regional impacts might exceed local impacts, as a higher number of small-ranged declining species are expected relative to the number of larger-ranged species that benefit from change. However, these approaches do not directly quantify the effects of increasing spatial scale on biotic homogenization^27^. We hypothesize that, despite regional variation in community responses, biodiversity change is less severe within single ecoregions (local scale) than when considered across multiple ecoregions (regional scale). We may thus be severely underestimating large-scale biodiversity change, which points to two key questions: (1) how do very large-scale biodiversity impacts compare to impacts within relatively homogeneous biogeographic units (ecoregions); and (2) what determines the degree to which regional-scale impacts exceed local-scale impacts?

**Fig. 1:**
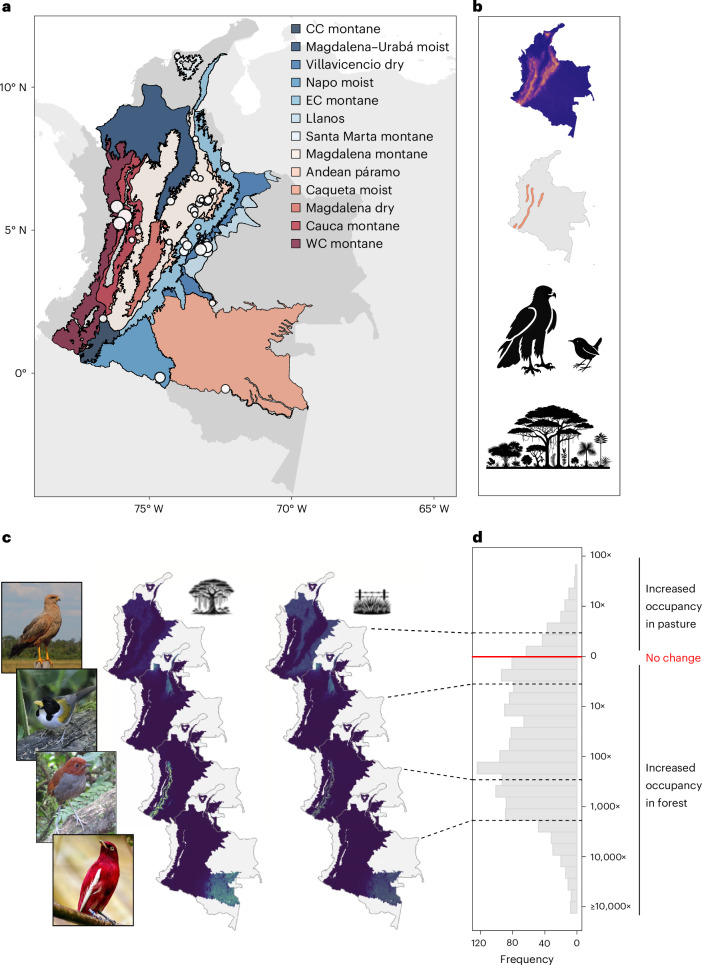
Methodological pipeline. Our pipeline combines field surveys and external data layers to yield detailed species-specific predictions of occupancy in forest and pasture, which enables computing species-specific sensitivities to forest conversion for any area at any scale, up to and including the entire study region. **a**, The locations of our 848 avian sampling points across Colombia, with larger circles representing larger concentrations of points. Background colours show the 13 biogeographic regions used in our analysis of regional versus pan-Colombian impacts. Portions of mainland Colombia that we exclude from our study region (see Methods) are rendered in grey. CC montane, Central cordillera montane; EC montane, Eastern cordillera montane; WC montane, Western cordillera montane. **b**, External data layers include geophysical data (elevation shown), species-specific biogeographic range information, functional traits and natural habitat associations. **c**, Field data (**a**) are integrated with external data (**b**) in a biogeographic multi-species occupancy-modelling framework to derive maps of occupancy probability in forest (left) and pasture (right), illustrated for four representative species, from top to bottom: savanna hawk (*Buteogallus meridionalis*), golden-winged sparrow (*Arremon schlegeli*), bicolored antpitta (*Grallaria rufocinerea*) and pompadour cotinga (*Xipholena punicea*). Colours in the map represent occupancy probability from near zero (dark blue) to high (yellow). **d**, The distribution of species-specific sensitivities to forest conversion is derived from species’ occupancy probabilities, here shown at the scale of the entire study region. Red line denotes no change in occupancy probability in response to land-use change. Photo credits: savanna hawk, D.P.E.; golden-winged sparrow, Tom Driscoll; bicolored antpitta, Mark Kosiewski; pompadour cotinga, Mike Goad (Pixabay).

We tackle these questions by empirically quantifying spatial scaling patterns of biodiversity loss via a large-scale avian field study contrasting natural habitat (mainly forest) and cattle pasture, conducted at a pan-national scale in the megadiverse country of Colombia. Our sampling used a space-for-time substitution, from which we upscale to generate a near-nationwide inference of the impact of converting locations from natural habitat to cattle pasture. Specifically, our fieldwork spans 848 forest and cattle pasture points matched for geographic and elevational proximity across 13 biogeographic regions (Fig. 1a), dwarfing the typical spatial scale at which field-based studies quantify biodiversity change. We sampled birds using a point count methodology with four visits across consecutive days, obtaining 24,981 detections of 971 bird species (excluding individuals detected flying over or at distances >100 m). We then modelled species-specific responses to forest conversion for 1,614 bird species (including 643 never-detected species) using a multi-species biogeographic occupancy modelling framework^28^ that accounts for imperfect detection while incorporating detailed range and trait information for all species (Fig. 1b and Supplementary Table 1). This allows us to predict within-range occupancy for each species at 2-km resolution across the 13 biogeographic regions and entire study region in both forest and pasture (Fig. 1c). We then quantified each species’ sensitivity to habitat conversion as the ratio of the number of cells it occupied if they were forested versus pasture (Fig. 1d and Supplementary Methods), generating a distribution of species’ sensitivity to habitat conversion, ranging from those that benefited to those that were strongly negatively affected by conversion. We used the 25th, 50th and 75th percentiles of this distribution to represent a subset of the community related to an assemblage of species that have the lowest, average and highest sensitivity to habitat conversion. The percentile-based species assemblages were used in downstream interpretation to understand how these different assemblages responded to habitat conversion.

## Results and discussion

### Severe losses at pan-Colombian versus ecoregion scale

**Fig. 2:**
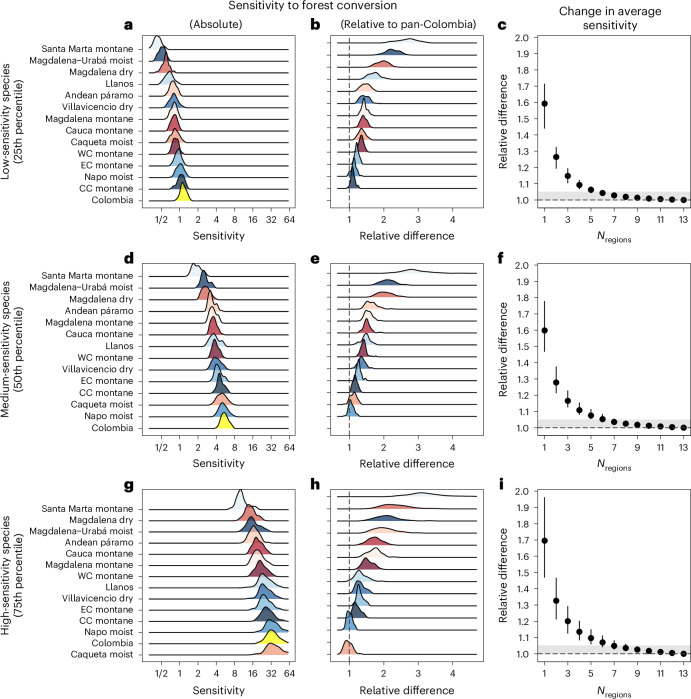
Avian community sensitivity to forest conversion. **a**–**i**, The distribution across species of regional avian community sensitivity to forest conversion (occupancy probability in forest divided by pasture), expressed in absolute terms (**a**,**d**,**g**) and relative to the pan-Colombia value (**b**,**e**,**h**), and the change in average sensitivity as regions are sequentially pooled together (**c**,**f**,**i**). **a**, **b** and **c** characterize the 25th percentile of the distribution across species (low sensitivity to habitat conversion), **d**, **e** and **f** the median (medium sensitivity), and **g**, **h** and **i** the 75th percentile (high sensitivity). In **b**, **e** and **h**, the vertical dashed line at 1 indicates parity between pan-Colombia sensitivity to habitat conversion and the sensitivity of the avian community within each sub-region; a relative difference of 2 indicates that pan-Colombia sensitivity is double that of a particular sub-region. **c**, **f** and **i** give average sensitivity to habitat conversion of collections of different numbers of subregions (point and 90% CI lines). The grey-shaded area represents the point where pooled-region sensitivity to habitat conversion is within 95% of the pan-Colombia score, with the dashed line at 1.0 indicating parity between pan-Colombia sensitivity to habitat conversion and the sensitivity of the avian community across pooled subregions.

The pan-Colombian community is far more sensitive to habitat conversion than the community present in any single region. Taking the relative median difference in sensitivity scores as our initial metric of biodiversity loss, losses at the pan-Colombian scale were 60% (credible interval (CI): 47–78%) more severe than single-region losses and 28% (CI: 21–38%) more severe than two-region losses (Fig. 2f). Species with higher sensitivity to habitat conversion (75th percentile; Fig. 1 and Supplementary Information) showed even greater losses at the pan-Colombian scale relative to losses at single-region (67%, CI: 49–89%) or two-region (30%, CI: 21–44%) scales (Fig. 2i), while species with lower sensitivity to habitat conversion (25th percentile) were similar in severity of losses to the median difference (Fig. 2c). Sampling of six to seven biogeographic regions was required before estimates were within 5% of the pan-Colombian value for species with low sensitivity (Fig. 2c), medium sensitivity (Fig. 2f) and high sensitivity (Fig. 2i) to habitat conversion. The majority of biodiversity field studies attempting to quantify the consequences of deforestation sample just one biogeographic region, or at best two to three^18,29^, probably leading to underestimation of the severity of deforestation-driven biodiversity loss, especially as findings are subsequently scaled-up using global meta-analysis^22,25,26^.

At the same time, we found considerable variation in the magnitude of impacts of habitat conversion on biodiversity across the 13 biogeographic regions (Fig. 2a,b,d,e,g,h). Communities in the central Cordillera montane forests, Napo moist forests, eastern Cordillera montane forests and Caquetá moist forests displayed greatest sensitivity to habitat conversion. These biogeographic regions are hyperdiverse lowlands or transcend major altitudinal gradients, resulting in high species packing, ecological specialism and low disturbance tolerance^30,31^. The biogeographic regions in which communities were least impacted by habitat conversion were those of Santa Marta, then the Magdalena–Urabá moist forests, Magdalena dry forests, Llanos and Andean páramos (moorlands), which are relatively species-depauperate areas (that is, isolated massifs and valleys, or very high altitude) and grassland–forest systems, where species are likely to be more disturbance- or edge-tolerant^32^. Differences in community-level responses to habitat conversion between biogeographic regions underscore that the choice of ecosystem in local-scale studies of land-use change impacts on biodiversity strongly affects study conclusions. While our spatial scaling patterns indicate low regional sensitivity to habitat conversion in certain biogeographic areas, this does not imply that forests are unimportant in these regions. For example, while the Sierra Nevada de Santa Marta (SNSM) was the least sensitive region in our analysis, reflecting a prevalence of forest-dwelling species that can frequently use pastures, its protected areas are among the most irreplaceable globally^33^ and its avifauna includes 24 endemic bird species, most of them overwhelmingly more common in forest than pasture^34^. Thus, the impacts of forest loss in SNSM for pan-Colombian biodiversity are severe, even though impacts in SNSM appear modest when compared with all other regions studied.

### High beta-diversity, distance and elevation drive losses

**Fig. 3:**
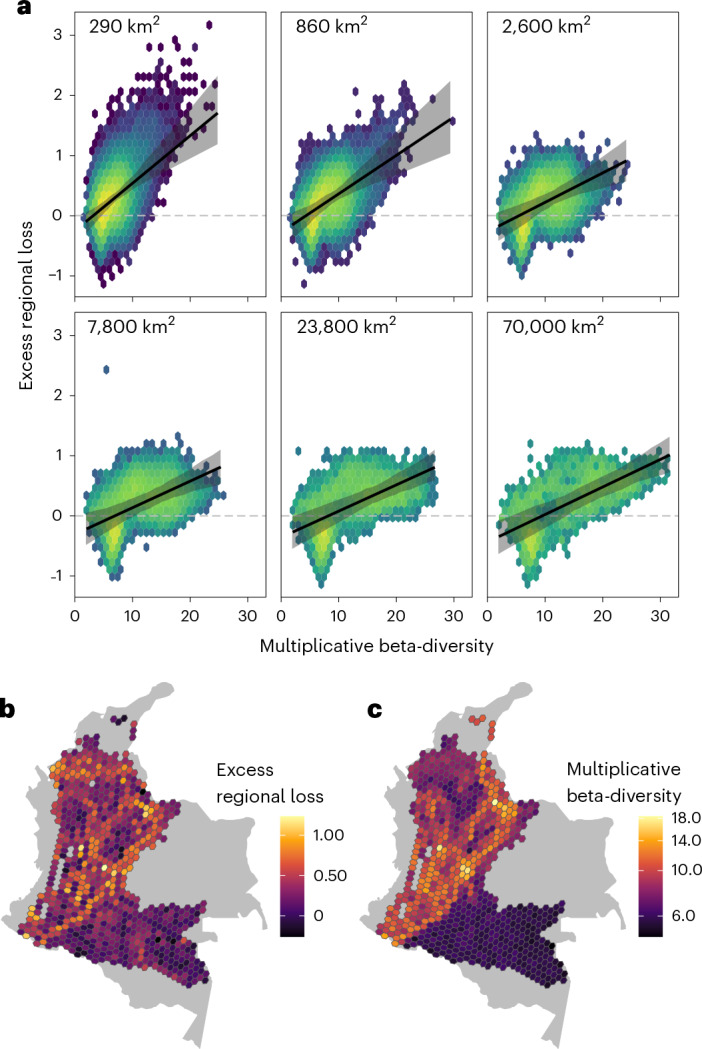
Differences between regional and local losses vary according to the beta-turnover of a region. **a**, Relationship between multiplicative beta-diversity and excess regional biodiversity loss at six regional spatial scales spanning more than two orders of magnitude in area. The excess regional loss is expressed as the log-ratio of regional to mean local (2-km pixel) loss, so zero represents no difference and one represents regional losses 2.7-fold worse than local. Here we show the median species loss metric (see Supplementary Fig. 5 for alternative measures). Background heatmaps represent the density of the underlying data points (yellow = very high density to blue = very low density). Grey ribbons are 90% CIs for the position of the line of best fit (that is, mean; black line) across posterior iterations. **b**, The underlying excess regional-over-local biodiversity loss at a region size of 860 km^2^. **c**, Multiplicative beta-diversity predicted from a representative posterior iteration at a region size of 860 km^2^. Each cell in these panels corresponds to one of the data points underlying the regression in the corresponding sub-panel of **a**. We randomly offset and rotated the hexagonal grid used in **b** and **c** at each posterior iteration.

Because biogeographic regions differ in their size and shape, we applied standardized hexagonal grids across the study region (Supplementary Fig. 2) to systematically investigate differences between regional and local community sensitivities (that is, the excess regional loss) in relation to beta-diversity. We found that when regional multiplicative beta-diversity is high, regional impacts of habitat conversion can average more than twice the severity of local impacts (Fig. 3), with progressively smaller discrepancies between regional and local impacts of habitat conversion in areas with lower beta-diversity. We computed excess regional biodiversity loss for each hexagon as the regional-scale loss metric (that is, percent decline of the median species, 25th percentile and 75th percentile) divided by the average local-scale (2-km pixel) loss, and regional multiplicative beta-diversity^35,36^ by dividing gamma diversity (species richness) of each hexagon by the mean alpha-diversity for 2-km pixels. In fact, regional multiplicative beta-diversity predicts excess declines in regional biodiversity via a relationship that appears to be largely independent of spatial scale (Fig. 3). While larger regions tend to have higher beta-diversity, the slope of the relationship between beta-diversity and excess regional impact is similar across at least two orders of magnitude of variation in hexagon area (from 290- to 70,000-km^2^ cells; Fig. 3). The tendency for both regions of high alpha-diversity (Fig. 2) and high beta-diversity (Fig. 3) to harbour particularly sensitive avifaunas suggests a connection between high species packing and disturbance sensitivity.

To unpack the drivers of beta-diversity that are associated with excess regional-scale impacts, we fitted generalized dissimilarity models (GDMs)^37^ to the bird communities at our sampling points, predicting compositional dissimilarity using geographic distance, elevation annual precipitation, and separation by montane or valley barriers for forest and pasture points separately (Supplementary Methods and Supplementary Fig. 3). In forests, compositional dissimilarity accumulated with increasing distance between sampling locations, with highly dissimilar communities found at forest points that were far removed from one another. Converting forest to pasture results in a collapse of this pattern, with negligible turnover found in pasture communities separated by more than 200 km, once other effects (for example, elevation) have been accounted for. The accumulation of disparity at spatial scales smaller than 200 km probably reflects small-scale variation between sites rather than biogeographic turnover of species: in our occupancy model, the random effect terms for spatial blocks corresponding to ~0.5-km and ~20-km scales were estimated with large variances (Supplementary Table 1), reflecting high small-scale heterogeneity that is decoupled from large-scale biogeographic variation.

**Fig. 4:**
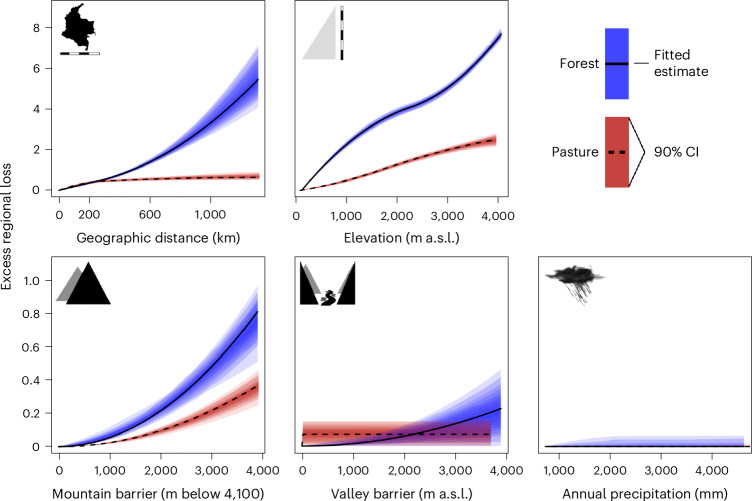
Impact of spatial and biogeographical gradients on community turnover. Community turnover across spatial and biogeographical gradients is substantially lower in pasture (red) than forest (blue). The panels indicate how the partial ecological distances vary along each respective gradient, based on a GDM fitted to the observed species assemblages at 848 cattle and pasture sampling points comprising 3,357 point visits (with the line of best fit (mean; black line) and 90% CI from a Bayesian bootstrap overlaid). Kilometres (km) represents the distance between pairs; other metrics represent the rate of turnover across the gradient for that variable (for example, m a.s.l.). For metres below 4,100, 0 represents being on the same side of the mountain barrier, increasing numbers represent being on the opposite side of the mountain and progressively farther down from the summit. Definitions of the mountain and valley barrier covariates are given in Supplementary Methods and Supplementary Fig. 3. Differences are based on Sørensen dissimilarities of observed communities, but an alternative set of results based on Simpson dissimilarities and detection-corrected data is given in Supplementary Fig. 6. Note that the *y*-axis scale changes between upper and lower panels.

The effect of elevation within forests had a similar magnitude to the effect of geographic distance, with substantially different communities found in forests at different elevations. As with geographic distance, cattle farming drives a striking flattening in the accumulation of heterogeneity along elevational gradients, particularly between 0–1,500 m above sea level (a.s.l.) and 3,000–4,000 m a.s.l. (Fig. 4 and Supplementary Fig. 6). This pattern reflects the expansion of non-forest species from Colombia’s low-elevation savannas and high-elevation grasslands into previously forested portions of the gradient. At middle elevations between 1,500 and 3,000 m a.s.l., the collapse of heterogeneity in pasture is less pronounced, in part because turnover within forest is substantially lower at these elevations than elsewhere. We found a much smaller role for mountain barriers, with increasing differences in both forest and pasture at lower elevations on alternate flanks of mountains, particularly so in forest (Fig. 4). There were no detectable roles for valley barriers and precipitation gradients (Fig. 4), although we did not sample the deciduous forests and drylands at the low extreme of the precipitation gradient.

### Conservation impacts of biotic homogenization at large scale

Given that 219 million hectares of tropical moist forest were converted pan-tropically between 1990 and 2019^38^, and the global rate of deforestation remains high, adequately quantifying the impacts of land-use change on biodiversity is critical. By synthetically analysing standardized samples spanning tremendous biogeographic variation, we identified major scale-dependent losses resulting from habitat conversion. This suggests that substantial biodiversity losses are overlooked by the preponderance of local-scale studies of land-use change and their aggregation into meta-analyses to draw regional-scale inference. Therefore, the failure to sample at sufficiently large spatial scales—especially across large geographical distances and elevational gradients—drives major underestimations of biodiversity loss in the Anthropocene.

In light of the ongoing drive for globally applicable metrics of biodiversity value to support biodiversity assessments and offsetting^39–41^, it is critical to develop measurements that accurately reflect value in the global biodiversity commons. Given that assessments of value are scale-dependent, our study makes the strong argument for the need to design monitoring schemes with embedded spatial structures, combined with metrics tailored to the appropriate regional scale of policy interest. This is essential to ensure that we are not underestimating or missing the large-scale outcomes of local land-use changes on regional biodiversity, although such a shift in study design, extent and metrics would require significant financial and time investment at a time of limited conservation resources. In turn, there is an urgent need for the development and widespread adoption of analytical frameworks and statistical tools capable of reliably delivering these metrics. Our analyses provide an example of such a framework that is applicable at scale even in the megadiverse tropics.

Our study highlights that unchecked land-use change across larger spatial scales drives biotic homogenization. While changes in local diversity are well known to alter local ecosystem functioning, the influence of biotic homogenization on biogeographic region to biome-scale ecosystem functioning is less understood. Studies conducted at large scales suggest a strong spatial component to variation in functional trait responses to climate change^42,43^, so we hypothesize that increasing biotic similarity will make biomes less resilient to environmental changes, increasing the risk of biome-wide tipping points. It is notable that the functional consequences of biotic homogenization in montane forests of the Americas show slower rates of change in functional attributes to climate change than communities in the lowland forests^43^.

We sampled low-productivity pastures, which represent the typical cattle ranching system in Colombia^44^ and sometimes contained isolated trees, hedgerows, small forest fragments or narrow riparian strips^45,46^. Relative to these traditional pastures, silvopastoral systems can triple bird species richness, double dung beetle species richness and increase ant abundance by 60% at local scale^47^. Whether the homogenizing effects of land-use change we observe in low-intensity cattle pastures could be at least partially mitigated by the retention of natural habitat features or silvopasture remains an important question. However, even in highly wildlife-friendly pasture in the Amazon, forest specialists are functionally absent^46^, suggesting that improving agriculture may be insufficient to counter biotic homogenization.

In conclusion, the failure to sample at sufficiently large spatial scales means that there is a systematic underestimation of the impacts of cattle farming. Our results further imply that biodiversity impacts of land-use conversion are underestimated in general, including any other crop or commodity that spans many ecoregions, including oil palm, soy, coffee and sugar cane^48–50^, as well as timber production from natural forests, including selective-logging practices^51,52^. The accrual of loss at larger spatial scales also highlights the value of preventing deforestation at any scale, as the conservation value even of small-scale protection increases when viewed through a regional lens as opposed to a local one. We thus need to implement effective protection across landscape-scale biogeographic variation combined with integrated conservation approaches that promote larger-scale retention of natural habitat. These strategies could involve major area-based targets (for example, 30% by 2030^29^; Half-Earth^53^), measures that focus on maintaining system-wide integrity (for example, Intact Forest Landscapes^54^) and the pan-tropical role of Indigenous communities in protecting lands from deforestation and degradation^55^. They should be underpinned by improved national to regional level land-use planning and by context-dependent targets that reflect the variation in community sensitivity to land-use change between biogeographic regions.

## Methods

### Study area and data collection

#### Study area

Colombia is a megadiverse tropical country^56^ whose biogeography is dominated by mountains. Three chains of the Colombian Andes and the isolated Santa Marta massif all reach elevations above 4,000 m a.s.l. and harbour distinctive endemic faunas on their slopes. These mountains structure climate and life zones, and together with the intervening Cauca and Magdalena inter-Andean valleys create imposing dispersal barriers between major biogeographic provinces. The east Andes, which extend north nearly to the Caribbean coast, are particularly significant as a biogeographic barrier for Colombia’s lowland fauna found to the east in the western Orinoco and Amazon. Additional salient features of Colombian biogeography include major lowland precipitation gradients and the abrupt transition between the savannas of the Llanos and the forests of Amazonia and the east Andes.

Across these diverse landscapes, pasturelands are the dominant agricultural land use in every major region of Colombia^57^, accounting for over 34 million hectares (30% of Colombia’s land area) and over 75% of all cleared forestland^57–59^. The importance of pasturelands (primarily for cattle) as a deforestation driver is expected to persist into the future^60^, posing a serious biodiversity threat^10^. The ubiquity of pastures across elevations and biogeographic regions allows us to study a single major land-use change across Colombia’s biogeographic variation.

We sought to capture as much biogeographic variation as possible with our fieldwork (Fig. 1). Fieldwork encompassed all three Andean cordilleras, the Santa Marta massif, and the lowlands of the Amazon, Llanos and Magdalena Valley, spanning elevations of 100–4,060 m a.s.l. (Fig. 1 and Supplementary Fig. 1). Fieldwork thus covered nearly all of Colombia’s major mainland biogeographic regions, yielding a dataset of sufficient breadth and depth to probe the spatial scaling of biodiversity change in a strongly heterogeneous region of the megadiverse tropics. We did not sample in the Pacific lowlands and northern dry forests due to COVID-19 travel restrictions, and eastern Llanos due to lack of extensive natural forest. When projecting species distribution models across Colombia, we therefore do not project to elevations below 1,100 m a.s.l. on the Pacific slope or in eight northern departments (northwestern Antioquia, Atlántico, Bolivar, Cesar, Córdoba, La Guajira, Magdalena, Sucre). We additionally refrain from predicting species distributions in the isolated Tacarcuna highlands of the Panamá border, the highest elevations of the SNSM (above 3,000 m a.s.l.), the eastern Amazon (where white-sand soils largely preclude cattle farming) and the eastern Llanos (where natural forest cover is minimal).

#### Site selection

We selected sites where high-quality forests or páramos (hereafter forest points) and cleared pastures (pasture points) exist within several kilometres of one another. Within sites, we installed clusters of three (very occasionally two) sampling points with 200 m minimum spacing. We selected points based on visual examination of current and historical imagery in Google Earth and options for access in extremely rugged terrain and amidst complex political situations. In total, we sampled 848 points in 287 clusters across Colombia.

In sampling broadly across a biogeographically complex study area, we took special care to ensure that our forest and pasture samples consisted of biogeographically comparable units. At middle elevations in the west Andes^45^, middle-to-high elevations of the east Andes^21,32^ and on the Llanos^61^, we sampled both forests and pastures intensively within well-defined biogeographic units^62^, and we took care to ensure that forest and pasture points had comparable geographic and elevational distributions. Elsewhere (that is, the central Andes, southern Andes, Santa Marta massif, Magdalena Valley, east Andean foothills, northern east Andes and Amazonia), we ensured comparability between forest and pasture samples by strictly matching paired clusters of forest and pasture points for elevation (<200 m difference) and geographic proximity (<21 km distance). The only exception to this rule came from one Amazonian site, where we surveyed eight forest clusters near the base of a sandstone outcrop, but found space to install only three pasture clusters. Therefore, we installed five additional pasture clusters near the base of a different sandstone outcrop, at similar elevations but 320 km to the north. The matched pairs design does not figure directly in our analysis, but ensures that our overall forest and pasture points sample from strictly comparable biogeographic units. We characterized the elevation of each point by using GPS-derived geospatial coordinates to extract elevations from the ALOS digital elevation model^63^, which we found to be substantially more reliable near some of our sampling points than other digital elevation models.

#### Bird surveys

Bird sampling was conducted in 2012–2013 and 2018–2019, avoiding the wet season months in locations with a strong precipitation seasonality (that is, not between mid-November and December, or late April to June). At each bird sampling point, one of five expert observers (J.B.S., S.C.M., D.P.E., J.J.G., O. Cortes) conducted four 10-minute, 100-m radius avian point counts on consecutive or nearly consecutive days. Observer O. Cortes refers to Oswaldo Cortes, who sampled only in the western Andes and conducted more than an order of magnitude fewer point count locations than J.J.G. in this region (O. Cortes = 9, J.J.G. = 109). Thus, for the purposes of estimating observer effects on detection (see below) we lumped O. Cortes and J.J.G. as a single observer, because O. Cortes conducted his point counts at the same time and landscapes as J.J.G. In total we conducted 3,357 point visits, representing 33,570 minutes of field observation.

We surveyed birds only in appropriate weather conditions (calm winds without steady rain) between the onset of the dawn chorus (roughly 30 minutes before sunrise) and 1230 h. At some sites above 3,000 m, where bird activity does not fall off sharply during the afternoon, we continued to conduct point counts to mid-afternoon. At a small minority of points, extenuating circumstances (illness, plane crash, guerrilla activity) restricted us to conduct only two or three visits, but this is dealt with in our analysis (see below).

We recorded sound continuously during avian point counts and identified unknown sounds with reference to comprehensive sound libraries including Xeno-canto and the Macaulay Library, and in consultation with leading experts in Colombian and neotropical birdsong (see Acknowledgements). We recorded abundance of individuals for each species in the field, but later turned these into presence–absence data to match species identified from the sound recordings, for which numbers of individuals could not be accurately estimated. Local vegetation cover was recorded at each cattle pasture point.

### Species list, ranges and traits

#### Baseline list

Our analysis explicitly handles both observed and never-observed species that potentially occur in the vicinity of our sampling points. To create a list of species that potentially occur near our points, we began by compiling a baseline species list for mainland Colombian birds. Our baseline list follows the taxonomy of HBW-BirdLife International version 4.0^64^ with one exception: on the basis of recent taxonomic revisions^65^, we treat BirdLife’s *Grallaria rufula* as consisting of four species in Colombia (*G. alvarezi*, *G. rufula, G. saturata* and *G. spatiator*). We harmonized an up-to-date species list for Colombia^66^ to the BirdLife taxonomy, and we removed species whose Colombian distribution is restricted to offshore islands or marine/coastal environments (for example, beaches, mangroves, lagoons, tidal mudflats) as well as species that occur in Colombia exclusively as vagrants^67,68^. We also removed all swifts (Apodidae) as they are essentially never observed perched. Finally, we removed the mallard (*Anas platyrhynchos*) because its introduced Colombian range is largely confined to urban habitats that do not figure in our sampling or analysis^66^.

#### Range maps and distance to range

Following the biogeographic multi-species occupancy model framework^28^, we used range maps to constrain species distributions to: (1) avoid predicting occupancy at biogeographically implausible locations; (2) avoid underestimating occupancy within a species’ range (because fitting to out-of-range points can underestimate in-range occupancy probability); and (3) reduce the computational costs of model fitting. We obtained digital range maps for all Colombian birds from Ayerbe-Quiñones^67–69^ (hereafter AQ) via the Instituto Alexander von Humboldt (http://biomodelos.humboldt.org.co/). We harmonized the AQ taxonomy to our baseline list, updating the maps as necessary to account for splits and lumps.

We used the range maps to construct a distance-to-range covariate for every species–point combination in Colombia. For out-of-range points, these are the (positive) distances to the nearest mapped range. For in-range points these are the (negative) distances to the nearest edge of the mapped range (excluding edges at coastlines or international borders). Colombia’s biogeography is dominated by four mountain ranges and the valleys between them and sometimes contain large rivers (that is, ríos Magdalena and Cauca). These topographic barriers imposed by Colombia’s mountains create extreme variation in how distance-to-range influences occupancy probabilities in Colombia’s birds. For example, lowland range extensions of 30 km are not noteworthy, but Andean range extensions of 30 km can cross major topographic barriers (indeed, montane elevations on opposite sides of the Cauca Valley are separated by as little as 10 km in places). Therefore, we divided Colombia into 11 topographic units based on mountain and valley barriers (Supplementary Fig. 1), and we computed the distance-to-range for each point based only on the mapped range in the same topographic unit as the point (that is, range that is not isolated from the point by a major topographic barrier). When there is no mapped range within a topographic unit, those species–point combinations are removed via biogeographic clipping. Note that these topographic units are not the same as the biogeographic regions that we use to analyse spatial scaling patterns (see below and Fig. 1). In fact, the topographic units and biogeographic regions strongly cut across one another and never share borders.

Our field data contained very few long-distance range extensions against the AQ maps, with distance-to-range of >160 km or crossing a topographic boundary for only 12 species (five of which we detected during a single expedition to the Tamá massif on the Venezuelan border). For these 12 species, we manually added range as described in Supplementary Appendix 1, yielding our final set of range maps (hereafter, the range maps). Because of the paired sampling design, these range additions do not bias forest/pasture comparisons. We manually added range for three reasons: (1) to ensure that the minority of species with poorly known ranges would not force the model to predict high out-of-range occupancy probabilities for the better-known majority of species; (2) to improve the accuracy of predicted occupancy probabilities for the 12 species in the vicinity of the range extensions; and (3) to enable biogeographic clipping at 160 km for computational efficiency in the occupancy model (see below)^28^.

#### Elevational ranges

Elevational ranges were derived from AQ. For species in our baseline list with no elevational range given by AQ (for example, where taxonomic treatments differ between lists), we incorporated information from additional references pertinent to Colombia and neighbouring countries^68,70–73^ (http://biomodelos.humboldt.org.co/, https://datazone.birdlife.org/). To reliably pool information on elevation–occupancy relationships across species, we linearly rescaled the elevations at all of our sampling points separately for each species, placing the elevations on a common scale where the species-specific minimum and maximum elevations given by AQ correspond to values of −1 and 1. We term these rescaled elevations species-standardized elevations.

Our field data exposed six severe deviations from AQ’s bird elevational ranges. These species were recorded at points falling outside a species-standardized elevational range of (−2.8, 2.8) (range chosen following data inspection; species outside this range are clear visual outliers), and we replaced their AQ ranges with elevations from ref. ^68^, which in each case caused all observations to fall within the (−2.8, 2.8) species-standardized elevation interval. It is not the case that AQ consistently reports tighter ranges than ref. ^68^; these cases seem to involve species for which AQ gives uncharacteristically conservative ranges.

#### Seasonality

For all migratory bird species, we compiled the approximate dates of presence in Colombia based on eBird data^74^ aggregated at the scale of Colombia supplemented by information from the field guide literature^68,75^. Our field-collected data exposed errors of omission in only two species, *Bartramia longicauda* and *Setophaga cerulea*, in each case of less than 7 days. We buffered the dates for these species to include the field-collected observations.

#### Final species lists and dataset size

We analyse only species that potentially occur in the spatio-temporal vicinity of our point counts, based on range maps, elevational ranges and migratory seasonality. In particular, we analyse only species with a distance-to-range of less than 160 km for at least one sampling point that falls within a species-standardized elevational range of (−3, 3) on an appropriate date. This process of biogeographic clipping (that is, excluding biogeographically implausible species–point pairs from analysis) yields large gains in computational efficiency without compromising the quality of the model fit^28^. These constraints yielded a final list of 1,614 bird species for analysis. Across our 848 sampling points, we retained 591,152 biogeographically plausible species–point pairs for analysis, of which 971 species and 15,543 species–point pairs had at least one detection.

#### Functional traits and range characteristics

We compiled information on multiple traits that we suspected a priori^76,77^ might influence species responses to pasture, including diet and body mass^78^, elevational range breadth and elevational range median^67,68^, migratory status^68^, range restriction at mountain barriers (that is, a species does not occur on both sides of the east Andes) and valley barriers^67,69^ (that is, a species does not occur both east of the Magdalena River and west of the Cauca River), and natural habitat associations^79^. To avoid injecting external information about responses to human disturbance, we did not incorporate any habitat-association covariates that directly reflect occurrence in anthropogenic habitat categories.

### Species distribution modelling

We modelled occupancy across the entire study area using a biogeographically constrained multi-species occupancy model (bMSOM)^28^ with range-map clipping at 160 km and elevational clipping to species-standardized elevations on the interval (−3, 3). The bMSOM improves on previous large-scale applications of community occupancy modelling by incorporating information from range maps, injecting species-specific trait information for never-detected species and reducing the computational costs of model fitting.

We leveraged our large dataset to fit a detailed data model. We modelled occupancy as a function of species-standardized elevation (linear and quadratic terms, estimated separately for species with elevational minima at zero versus greater than zero), a monotonic effect^80^ of distance-to-range, land use (forest or pasture), biogeographic and functional species traits (Supplementary Table 1), and the interactions between land use and all species traits. To leverage phylogenetic information and account for species-level variation, we included random intercepts grouped by species and family, a random slope for elevation by species, and random slopes for land use by species and family. To address spatial autocorrelation, we included random intercepts grouped by species-by-cluster and species-by-subregion. We included the subregion effects after fitting the model with just cluster effects and detecting unmodelled spatial autocorrelation that was strongest at the 20-km scale via a posterior predictive check (see below). Therefore, we grouped points into subregions at the 20-km scale.

We modelled detection as a function of observer, time after sunrise, land use and five species traits (Supplementary Table 1). We included random intercepts grouped by species, family and species-by-observer. We included random coefficients for time after sunrise by species and for land use by species and family.

#### Prior specification

Overly vague priors on logit-scale parameters are known to cause problems in occupancy models for modest-sized datasets, as they induce highly informative pushforward densities on the probability scale, concentrated at probabilities near zero and one^81,82^. This phenomenon becomes even more extreme in models with a large number of covariates^83^. Our massive dataset probably avoids some of the resulting problems, but we nevertheless took care to specify principled priors, based on extensive personal experience^8,84^ and literature on the structure and organization of neotropical bird communities^84–87^, trait-based predictors of avian sensitivity to deforestation^26,76,77^, and trait-based predictors of variation in avian detectability^88^. To ensure that covariate relationships are driven by the data and not the priors, we use Gaussian zero-centred priors for all coefficients. Thus, our priors constrain the effect sizes to reasonable values but inject no information about the directionality of the effects, and any tendency for coefficients to have non-zero marginal posterior distributions is not due to the prior. We chose weakly informative priors for both coefficients and scale parameters (that is, random effect standard deviations) that understate the certainty of our prior knowledge while simultaneously avoiding pushforward densities on the probability scale that are outlandishly concentrated towards extreme probabilities near zero and one. We provide a detailed description of our prior selection in the supplementary information accompanying ref. ^28^, where we develop the biogeographic multi-species occupancy-modelling framework that we use here. We provide a summary of our priors in Supplementary Table 1.

To ensure that pushforward densities for different species–point combinations are not unduly influenced by our choices of reference categories for dummy-coded binary predictors, we coded all binary predictors as −1/1 rather than 0/1 (ref. ^89^). In addition to ensuring equivalent prior pushforward densities across different combinations of predictors, this coding places binary predictors and standardized continuous predictors on the same scale^90^.

#### Model fitting and criticism

We fitted the occupancy model in Stan^91^ via R package flocker^92^, which provides an occupancy-modelling front end for R package brms^93^. We ran four chains for 1,000 warmup iterations and 1,000 sampling iterations each. All chains ran without post-warmup divergences and yielded estimated fractions of missing information greater than 0.88. Across all four chains, all parameters yielded R-hat (split, folded, rank-normalized) of less than 1.02 and bulk effective sample size greater than 100.

Our data are sufficiently strong to robustly estimate the global model and constrain species-specific occupancy estimates despite some covariates having small or uncertain effects. Therefore, we did not perform model selection. However, our final model was developed via a process of model refinement in response to posterior predictive checking (see below). To promote transparent science and expose ‘researcher degrees of freedom’^94^ in our approach that would otherwise remain hidden, we document our model-building process in detail in Supplementary Appendix 2.

We assessed model adequacy with multiple posterior and mixed predictive checks^95^ tailored to detect specific forms of misspecification. Because our model is fitted to over 2 million data points, we have tremendous power to detect mild forms of misspecification. Therefore, our focus is not on ensuring that we ‘pass’ the checks, but rather on guiding model development to arrive at an adequate model without obvious avenues for further improvement. We describe these checks in detail in Supplementary Appendix 3.

### Cross-scale metrics of biodiversity impact

For both our ecoregion-based analysis and our grid-based analysis, we require a metric of biodiversity impact that captures the biodiversity change associated with forest conversion to pasture. Crucially, this metric must straightforwardly capture the relative conservation value of forest and pasture, but must also be straightforwardly comparable across spatial scales. For example, raw population size of a species of conservation concern is not a useful metric, as it will naturally tend to increase with increasing spatial scale, even in the absence of any noteworthy scaling dynamics.

The natural metrics for our use involve characterizing the distribution across species of percent changes in occupancy between forest and pasture. For example, over any spatial area we can assess the ratio of forest occupancy to pasture occupancy for the median species (among all species present in the area), and then ask whether and how this median varies systematically with spatial scale. To provide a more complete view of how the distribution of species-specific abundance ratios varies across spatial scales, we report not only the median but also the 25th and 75th percentiles. If communities tend to accrue disproportionately more losers than winners with increasing spatial scale, then these metrics will report greater biodiversity impacts at large scales compared with small scales.

Using our species distribution models, range maps and digital elevation model, we predicted species-specific occupancy in forest and pasture landscapes on a 2-km spatial grid across Colombia. We made these predictions for hypothetical forest and pasture landscapes at each location irrespective of current land use. Because one 2-km grid cell contains sixteen 500-m cells, each corresponding to the scale of one of our sampling clusters, we computed the predicted occupancy sampling sixteen times from the cluster-level random effect and then averaging on the back-transformed probability scale.

From these predictions, it is straightforward to calculate the proportional change in total occupancy (expected number of total forest points occupied divided by total pasture points occupied) for any species in any region of any size. Thus, we can compute the metrics laid out above by taking these ratios for all species in the regional species pool and finding the 25th, 50th and 75th percentiles. However, a challenge remains, namely that of selecting the set of species we use to represent the regional species pool. In particular, we wish to avoid results in which the distribution of population changes is dominated by a huge number of species that are absent or vanishingly rare in the region. On the other hand, we want to allow our metrics of biodiversity impact to reflect the fates of highly localized species and/or constitutively rare species that are characteristic of the region.

Therefore, we classified species as potentially present at the pixel scale based on thresholds of occupancy probability. Results in the main text are based on species pools defined as all species that reach an occupancy probability of at least 0.2 on at least one relevant 2-km pixel. Thus, regional species pools are species that reach the threshold occupancy probability on at least one pixel within the region. Local species pools are species that exceed the threshold locally. In Supplementary Results, we additionally present results for alternative thresholds of 0.1 and 0.3. Note that because of the relatively large variance associated with subregion and cluster effects in our model, even constitutively rare species can be expected to exceed occupancy probabilities of 0.2 at some points. For example, in a single homogeneous subregion (20 km × 20 km) a species with the following mean occupancy probabilities would be expected to meet our threshold in at least one 2-km pixel: mean occupancy of 0.03 would be expected to meet the threshold probability of 0.1; mean occupancy of 0.09 would be expected to meet the threshold probability of 0.2; and mean occupancy of 0.15 would be expected to meet the threshold probability of 0.3. In regions spanning multiple subregions, the large fitted variance of the subregion effects would come into play, depressing these numbers still further.

We chose these thresholds to ensure, on the low end, that we include constitutively rare species within our metrics of biodiversity loss and to reflect, on the high end, values at which a well-powered local-scale study of 100 points (50 in forest and 50 in pasture, each one exhaustively sampled to detect all species that are present) would be expected to obtain enough detections to make single-species inference about the relative value of forest and pasture.

### Quantifying multi-region impacts

To quantify the sensitivity to habitat conversion of the community in a particular region of Colombia, as well as across the union of regions (the ‘Colombia-wide’ scale), we calculate the community that is present in each region, and then quantify how these communities respond to forest conversion. To establish how a given species responds to forest conversion, we first calculate the ratio of the number points that a species is predicted to be on if all points were forested to the number of points if all points were pasture. Following this, we ask which species are present in which region’s community based on the thresholding above. Identifying the species pool associated with each region (and their union, for the Colombia-wide pool) results in a distribution of species’ sensitivity to habitat conversion, ranging from species that benefit from forest conversion to species that are strongly negatively affected by forest conversion. To generate measures of the full distribution of sensitivity to habitat conversion, we use three metrics to quantify the response of the community: the effect of habitat conversion on the 25th, 50th and 75th percentiles of sensitivity. We carry out this calculation for 100 draws from the posterior distribution, and present this posterior distribution in Fig. 2.

To assess how the sensitivity to habitat conversion of a particular region’s community compares with that of the community present across all regions (Colombia-wide), we then calculate each region’s sensitivity to habitat conversion relative to that across all regions as the ratio of the two. A value of one indicates that there is no difference in the sensitivity to habitat conversion of a region’s community from that present across Colombia as a whole, less than one indicates that the region is less sensitive, and more than one, more sensitive. As before, we carry out this calculation for 100 draws from the posterior distribution, and present this posterior distribution in Fig. 2.

Lastly, to establish how many regions we need to pool together to reach a similar level of sensitivity to habitat conversion as we see across all regions, we randomly generate 1,000 different sequences of regions, each of which starts with one region and then sequentially adds more regions until all 13 regions are present. We then iteratively work through each growing region list, adding to the species pool and updating the sensitivity to habitat conversion metrics. For each posterior draw and each sequence we then calculate the sensitivity to habitat conversion of the community relative to that observed across all regions. We then calculate the average relative sensitivity to habitat conversion by averaging across all sequences, and then summarize this to a mean and 90% CI, which is presented in Fig. 2.

### Quantifying predictors of spatial scaling

#### Beta-diversity as a predictor of excess regional impacts

To assess the spatial scaling of the relative conservation value of forest and pasture, we aggregated on hexagonal grids of various sizes^96^ and computed the conservation value over each hexagonal grid cell. We consider grid cells ranging from 290 km^2^ to 70,000 km^2^ (Supplementary Fig. 2). In each region, we compute our cross-scale impact metrics both at the scale of the whole cell and for each 2-km pixel inside the cell. We perform these computations iteration-wise over the occupancy model posterior, randomly offsetting and rotating the hexagonal grid at each rotation to avoid any artefacts associated with particular grid configurations. Note that it is because of these offsets and rotations that we display only a single representative posterior iteration (rather than an average over posterior iterations) in Fig. 3b,c. To ensure that we analyse cells of approximately constant size, we only include hexagonal cells that are at least 60% overlapped by our study area.

We compute the excess regional biodiversity loss as the regional-scale loss metric (that is, the percent decline of the median species, the 25th percentile and the 75th percentile) divided by the average of the local-scale (2-km pixel) metrics within the region. We then compute multiplicative beta-diversity^35,36^ based on species richness (using the thresholding approach discussed above to define the gamma diversity of the grid cell), and we use ordinary least squares to find the line of best fit in a regression of the logarithm of excess regional biodiversity loss against beta-diversity. We propagate uncertainty into this regression by re-computing the line of best fit iteration-wise over the posterior. Because the grid comprehensively covers the spatial domain under study, we employ ordinary least squares only to find the line of best fit through our sampled grid cells, not to make inference about some hypothetical larger population of cells. Thus, we are uninterested in the uncertainty in the fitted linear regression parameters within each posterior iteration. The uncertainty in our line of best fit comes from posterior uncertainty in the beta-diversities and excess regional losses across our study region, and this uncertainty is propagated fully.

#### Drivers of turnover in forest versus pasture

To explore the patterns of biotic homogenization that drive the collapse of biodiversity value at large spatial scales, we fitted GDMs^37^ to our detection-corrected point count data separately in forest and pasture. While analyses of pairwise dissimilarity cannot directly reveal the spatial scaling of biodiversity loss^27,97^, they can provide post hoc insights into the mechanism and pattern underlying the scaling phenomena that we have directly quantified through other approaches.

We used the Sørensen dissimilarity and the Simpson dissimilarity (that is, the turnover component of the Sørensen dissimilarity^36^) as response variables in our GDMs, and we modelled these dissimilarities based on geographic distance, elevation^63^, annual precipitation^98^, and measures of the degree to which points are separated by valley and mountain barriers. We define these two barrier measures as follows.

##### Valley barriers

We assigned all sampling points to one of four mountain ranges: the east Andes (including Amazonia and the Llanos), the central Andes, the west Andes and the SNSM. Point pairs on the same mountain range were assigned a distance of zero; they are not isolated from one another by valleys. Point pairs on different mountain ranges were assigned a distance corresponding to the elevation of the lower point, as valley barriers should be increasingly important in structuring dissimilarities in higher-elevation avifaunas (Supplementary Fig. 3).

##### Mountain barriers

We measured mountain barriers with respect to the crest of the east Andes, which represents the major biogeographic divide between the *cis*- and *trans*-Andean avifaunas. Point pairs on the same side of the east Andes were assigned a distance of zero. Point pairs on opposite sides were assigned a distance corresponding to 4,100 (the highest elevation sampled, and higher than most mountain passes in the east Andes) minus the elevation of the higher point, as montane barriers should be increasingly important in structuring dissimilarities in lower-elevation avifaunas (Supplementary Fig. 3).

We fitted our GDMs with a negative-exponential link function via R package gdm^99^ using three I-spline basis functions per predictor with knots at the minimum, median and maximum predictor values^37^. We used the Bayesian bootstrap^77^ to account for uncertainty in the fitted GDMs^78^, based on 400 bootstrap replicates. We fitted these GDMs both based on our raw point count data without correction for imperfect detection and based on modelled detection-corrected data. When using detection-corrected data, we fully propagated uncertainty in the occupancy model by fitting a single bootstrap replicate to each of 400 draws from the detection-corrected posterior.

### Software and data

We performed all data manipulation and analysis in R^100^, Stan^91^ and Google Earth Engine^101^. We constructed and fitted our occupancy models using R package flocker^92^, which we purpose built for this work to provide a front end to fit occupancy models in Stan via R packages brms^93^ and cmdstanr^102^.

### Reporting summary

Further information on research design is available in the Nature Portfolio Reporting Summary linked to this article.

## Supplementary information

Supplementary Information Supplementary Figs. 1–13, Table 1, Methods, Results, Appendices 1–3 and References.

Reporting Summary

## Data Availability

Data are available via Zenodo at 10.5281/zenodo.15318728 (ref. ^103^).

## Code availability

Code to reproduce all analyses is available at https://github.com/jsocolar/colombiaBeta.
