# Supplementary material for: Tropical biodiversity loss from land-use change is severely underestimated by local-scale assessments
Source: Nat Ecol Evol. 2025 Jul 22;9(9):1643–55. doi: 10.1038/s41559-025-02779-4 (PMC12420384; doi:10.1038/s41559-025-02779-4)
Supplement: Supplementary file 1 — Supplementary Figs. 1–13, Table 1, Methods, Results, Appendices 1–3 and References. [file 41559_2025_2779_MOESM1_ESM.pdf]

nature ecology & evolution

Supplementary information

https://doi.org/10.1038/s41559-025-02779-4

Tropical biodiversity loss from land-use change is severely underestimated by local-scale assessments

In the format provided by the authors and unedited

Supplementary Methods

Supplementary Figure 1

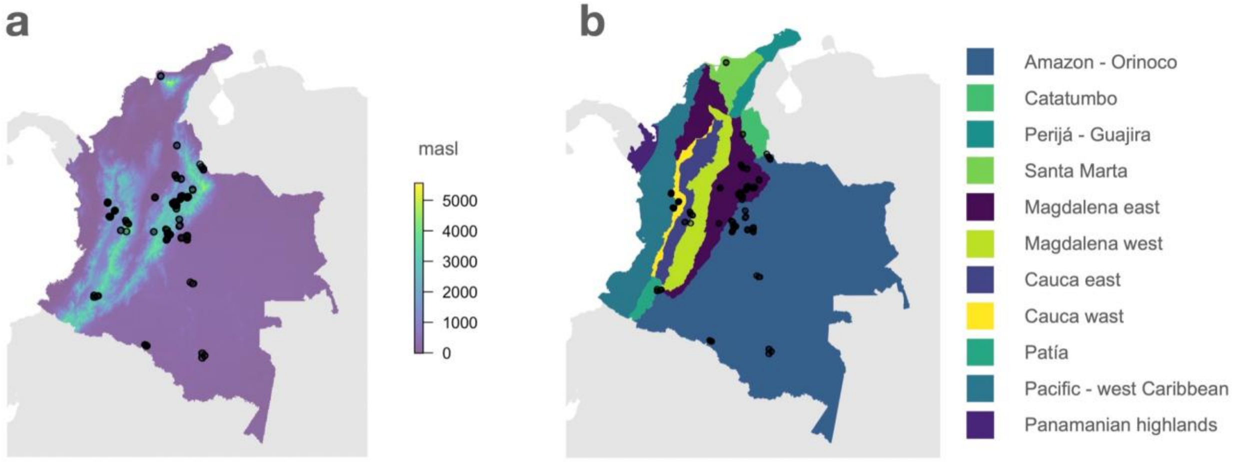

Supplementary Figure 1 Sampling points overlaid on maps of a) elevation and b) topographic units. The topographic units are: i) Amazon-Orinoco drainage; ii) Catatumbo drainage; iii) Perijá Mountains and Guajira Peninsula (east of the César and Ranchería Rivers); iv) Sierra Nevada de Santa Marta (east of the Magdalena River and west of the César and Ranchería Rivers); v) eastern and northern Magdalena Valley (east of the Magdalena River and south of the César River, plus west of the Magdalena River and north of the Cauca River); vi) western Magdalena Valley (west of the Magdalena River and south of the Cauca River); vii) eastern Cauca Valley (east of the Cauca River); viii) western Cauca Valley (west of the Cauca River); ix) Patía valley east of its water gap through the western Andes, x) Pacific slope and western Caribbean lowlands (west of the Magdalena/Cauca basin but excluding the highlands along the Panamanian border and the Patía valley east of its water gap through the western Andes); xi) Panamanian borderland highlands including Cerro Tacarcuna.

Supplementary Figure 2

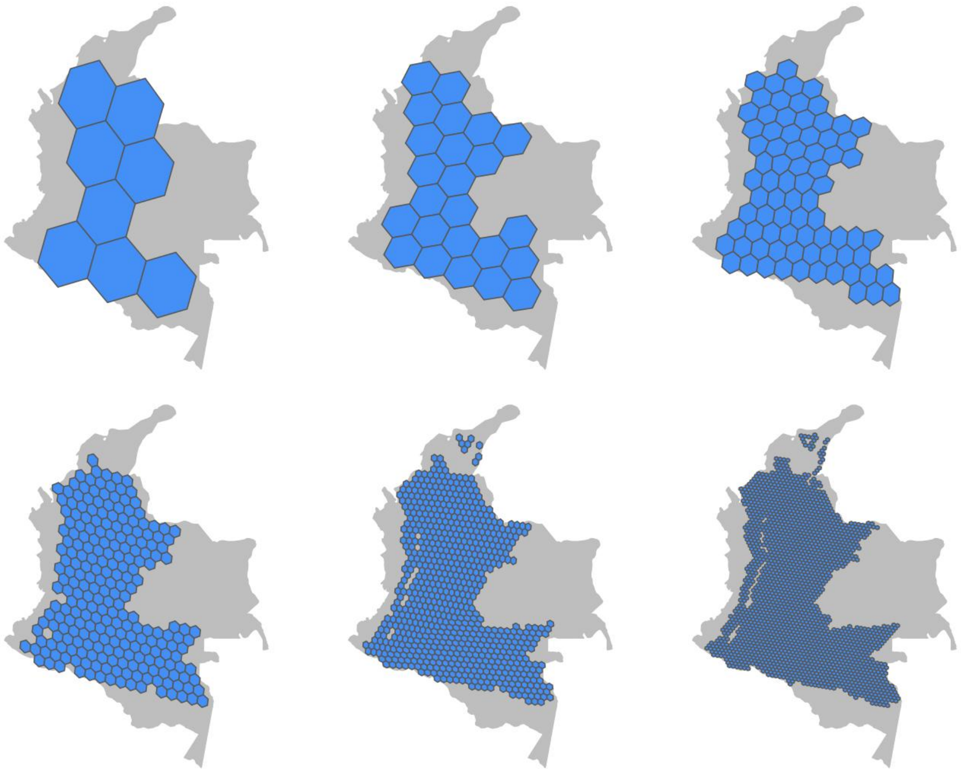

Supplementary Figure 2 Example hexagonal grids of different sizes (70000 km^2, 23000 km^2, 7800 km^2, 2600 km^2, 860 km^2, 290 km^2) used to study excess regional losses. These grids are an example; the actual grids used are randomly offset and rotated at each posterior iteration to ensure that results do not contain artifacts due to the particular choice of grid. Only grid cells overlapped at least 60% by our prediction area (see main text Figure 1) are included.

Supplementary Figure 3

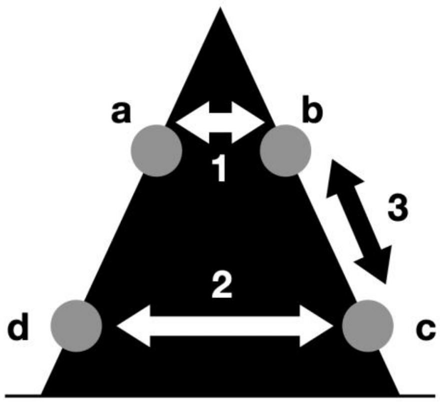

Supplementary Figure 3: The GDM predicts pairwise dissimilarity between points using additive terms (on the link scale) including terms for elevation and biogeographic barriers. The ecological distance associated with a barrier is assumed to decrease (mountain) or increase (valley) with the elevation of the points. For example, in the context of a mountain barrier the ecological distance (**1**) between points **a** and **b** is expected to be smaller than the distance (**2**) between **c** and **d**, because the high-elevation fauna will be more likely than the low-elevation fauna to cross the crest. The ecological distance between **a** and **c** is modeled as the sum of the barrier-distance (**1**) and the elevation-distance (**3**). Thus, the barrier-distance depends on the elevation of the higher point (or for a valley barrier, the lower point).

Supplementary Results

We regenerated Figure 2 from the main text for alternative values of the threshold for inclusion in the regional community (see Supplementary Methods: Cross-scale metrics of biodiversity impact). The main text reports results based on a threshold of 0.2; here we also show 0.1 (Supplementary Figure 4a) and 0.3 (Supplementary Figure 4b). The results are insensitive to the choice of threshold.

We likewise regenerated Figure 3 from the alternative thresholds, but also for alternative percentiles of the distribution of species abundance changes. The main text reports results based on a threshold of 0.2 and based on the median species in the community. Here, we present results based on alternative thresholds of 0.1 (Supplementary Figure 5c-e) and 0.3 (Supplementary Figure 5f-h). Results are strikingly consistent across alternative thresholds. We also show results based on the low-sensitivity fraction of the community (species at the 25th percentile of forest-divided-by-pasture occupancy ratios; Supplementary Figure 5a,c,f), the median species (Figure 3; Supplementary Figure 5d,g), and the high-sensitivity fraction of the community (species at the 75th percentile of forest-divided-by-pasture occupancy ratios; Supplementary Figure 5b,e,h).

Results are broadly consistent across the range of species sensitivities, with the distribution of sensitivities in the regional pool shifted to approximately double the sensitivity of the local pool at high beta-diversity. We do note, however, that the high-sensitivity results (species at the 75th percentile of forest-divided-by-pasture occupancy ratios) for threshold probabilities of 0.1 show a nonlinearity wherein our predicted y-intercept at zero beta-diversity is negative. This indicates a nonlinearity because at zero

beta-diversity the local and regional communities are identical, and so the true y-intercept must be zero. This nonlinearity appears to be related to the large number of Amazonian points in our sample where local losses are estimated to be more severe than regional losses for 75th percentile of species. Given that Amazonian avifaunas are characterized by many constitutively rare species[89] and are highly disturbance-sensitive[21], one possibility is that sparse data led us to mildly underestimate the sensitivities of less common Amazonian species that are regularly absent from any given location, leading to artifacts where regional scales have underestimated sensitivity relative to local scales. Consistent with this possibility, this artifact appears strongly only when using our lowest probability threshold for species inclusion.

Finally, we regenerated Figure 4 using alternative data sources and dissimilarity metrics. Figure 4 is based on Sorensen dissimilarities in our raw point count data. In Supplementary Figure 6, we show results based on Sorensen dissimilarities in our detection-corrected modeled occupancy data (Supplementary Figure 6a), and Simpson dissimilarities in both our raw data (Supplementary Figure 6b) and detection-corrected data (Supplementary Figure 6c). The Simpson dissimilarity reflects the component of the Sorensen dissimilarity that is due to turnover rather than to variation in species richness between samples[27]. The patterns that we observe in these alternative specifications produce a result that is qualitatively the same: geographic distance and elevation are the major drivers of community turnover, and are flattened in pasture relative to forest.

Supplementary Figure 4

(a) Inclusion probability threshold of 0.1

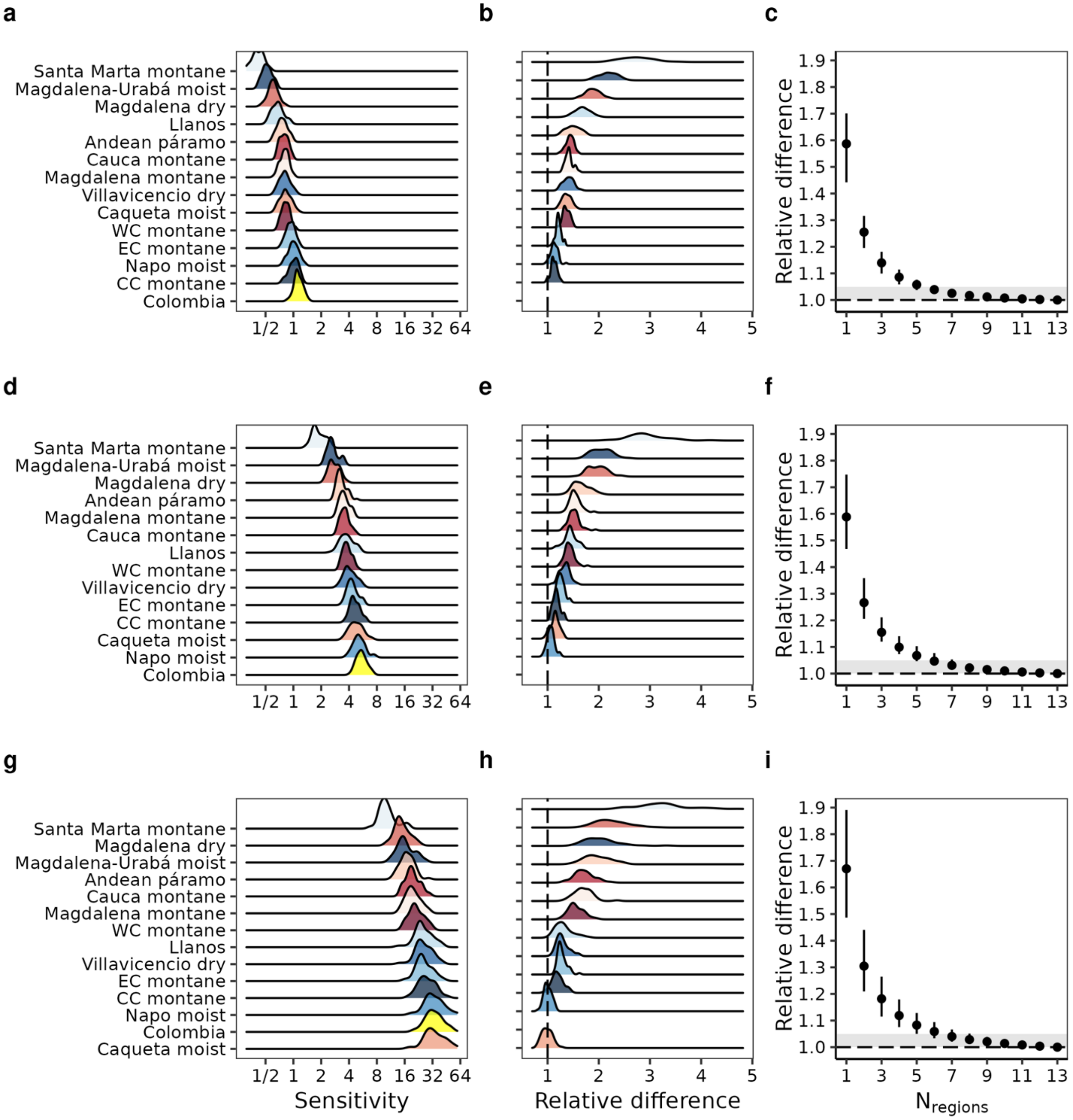

(b) Inclusion probability threshold of 0.3

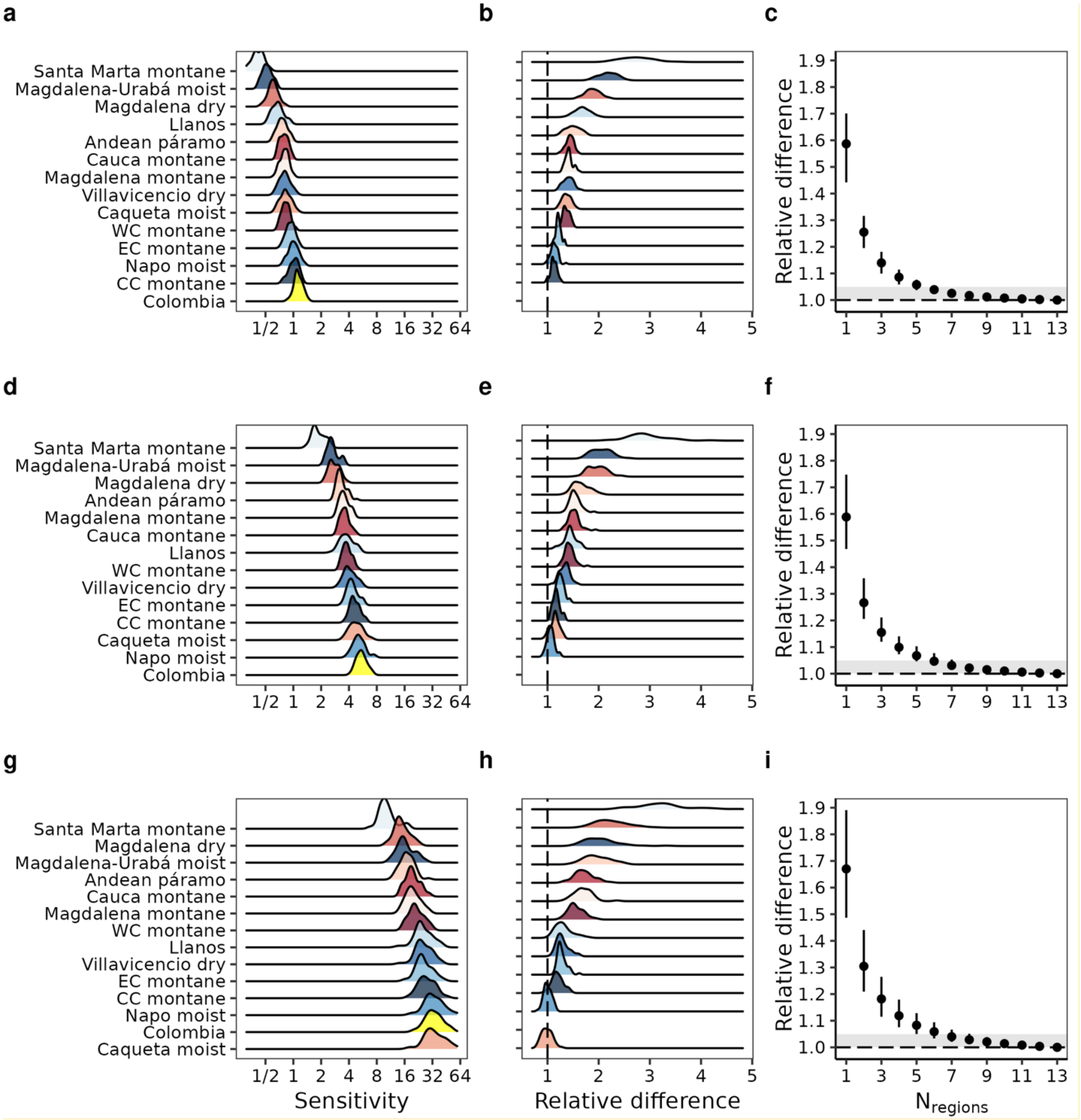

Supplementary Figure 4: Sensitivity to conversion between ecoregions figure using alternative thresholds of 0.1 and 0.3. The distribution across species of regional avian community sensitivity to forest conversion (occupancy probability in forest divided by pasture), expressed in absolute terms (panels a, d, and g), and relative to the pan-Colombia value (panels b, e, and h), and the change in average sensitivity as regions

are sequentially pooled together (panels c, f, and i). Panels a, b, and c characterize the 25th percentile of the distribution across species (low sensitivity), panels d, e, and f the median, and g, h, and i the 75th percentile (high sensitivity). Panels c, f, and i give average sensitivity of collections of different numbers of subregions (point and 90% credible interval lines). The gray-shaded area represents the point where pooled-region sensitivity is within 95% of the pan-Colombia score, with the dashed line at 0 indicating parity between pan-Colombia sensitivity and the sensitivity of the avian community across pooled sub-regions. Results are shown for two alternative regional threshold probabilities for inclusion in the regional community (10% and 30%), while a threshold of 20% is used in the main text (see Supplementary Methods: Cross-scale metrics of biodiversity impact).

Supplementary Figure 5

a) Low-sensitivity species (25th percentile species of forest-divided-by-pasture occupancy); inclusion probability threshold 0.2

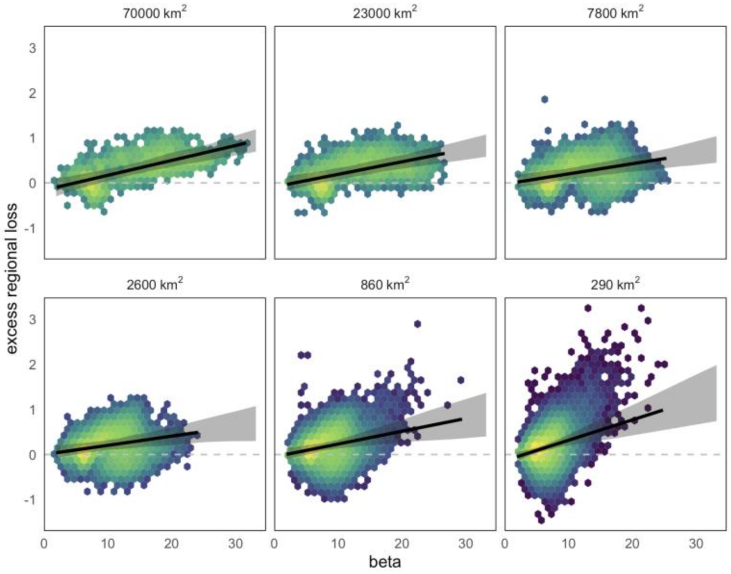

b) High-sensitivity species (75th percentile species of forest-divided-by-pasture occupancy); inclusion probability threshold 0.2

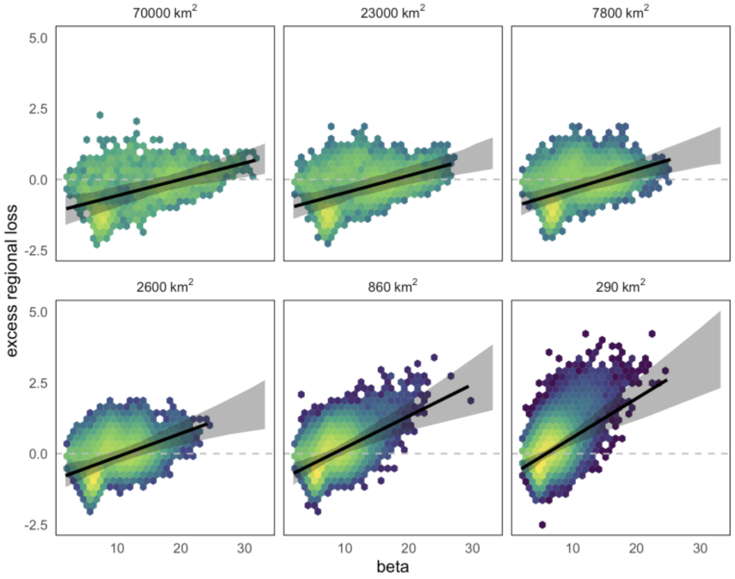

c) Low-sensitivity species (25th percentile species of forest-divided-by-pasture occupancy); inclusion probability threshold 0.1

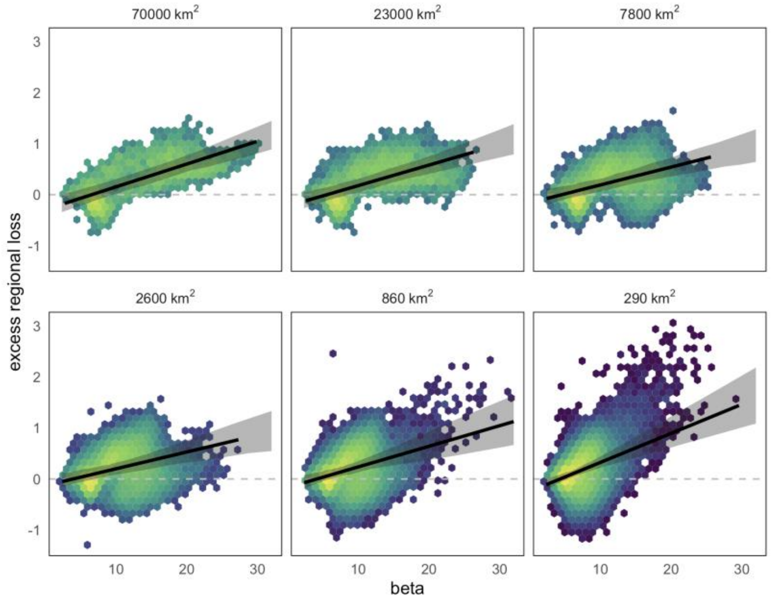

d) Median-sensitivity species (50th percentile species of forest-divided-by-pasture occupancy); inclusion probability threshold 0.1

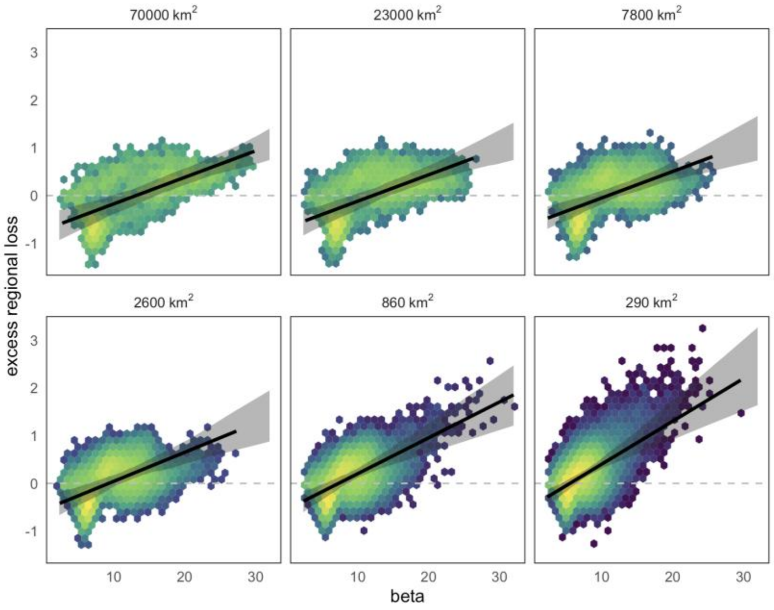

e) High-sensitivity species (75th percentile species of forest-divided-by-pasture occupancy); inclusion probability threshold 0.1

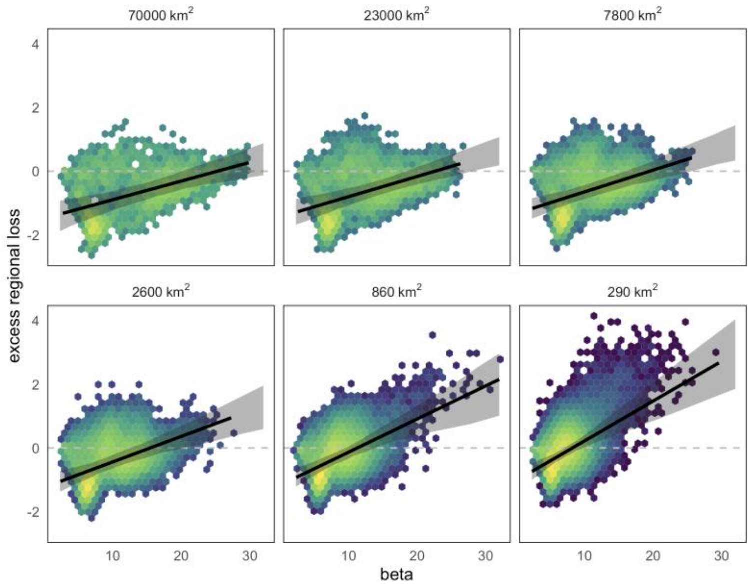

f) Low-sensitivity species (25th percentile species of forest-divided-by-pasture occupancy); inclusion probability threshold 0.3

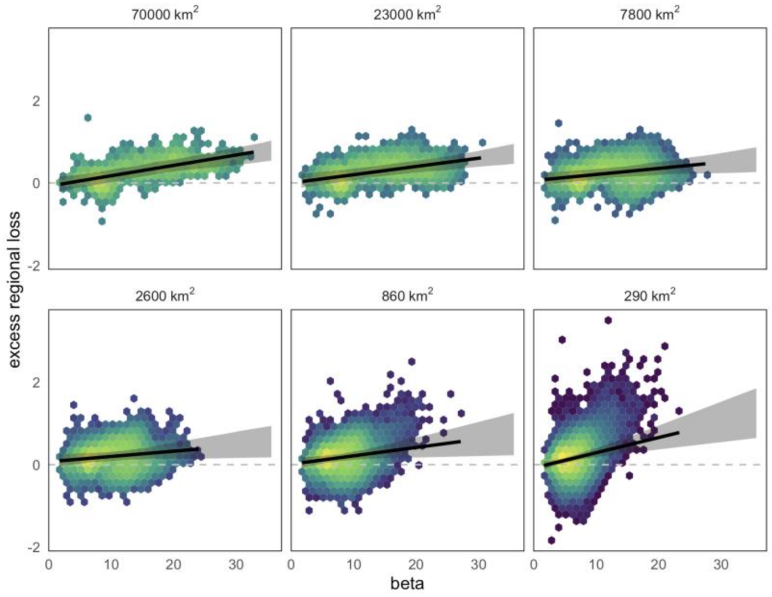

g) Median-sensitivity species (50th percentile species of forest-divided-by-pasture occupancy); inclusion probability threshold 0.3

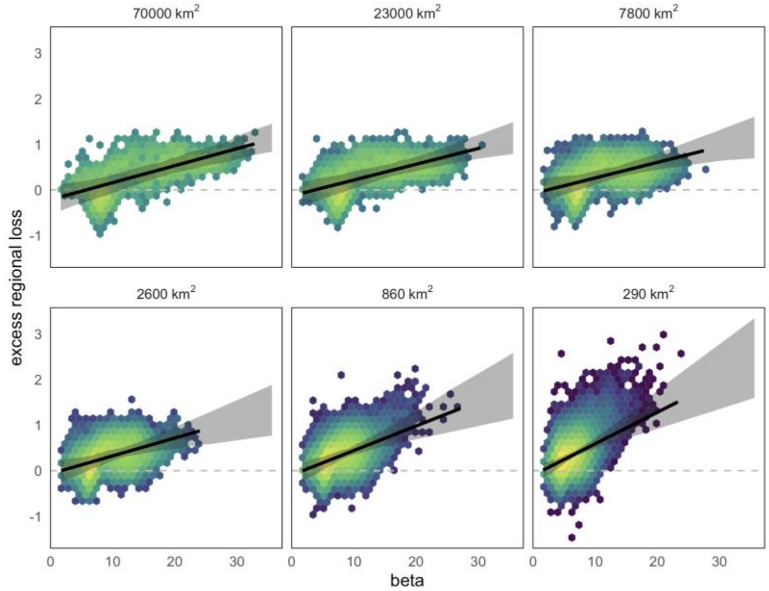

h) High-sensitivity species (75th percentile species of forest-divided-by-pasture occupancy); inclusion probability threshold 0.3

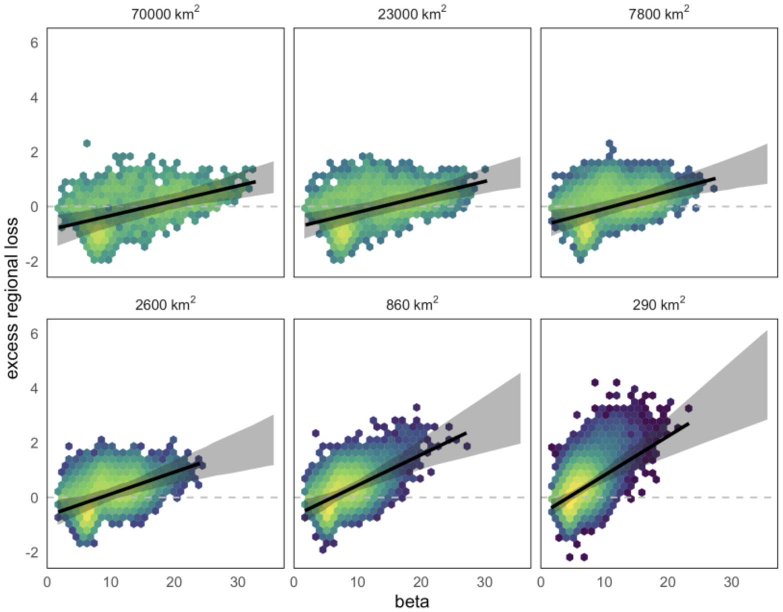

Supplementary Figure 5 Relationship between multiplicative beta-diversity and excess regional biodiversity loss at six different regional spatial scales spanning >2 orders of magnitude in area. The excess regional loss is measured as the log-ratio of the median and quartiles of the species sensitivity at the regional scale versus the local scale. Results are shown for three alternative regional threshold probabilities for inclusion in the regional community (see Supplementary Methods: Cross-scale metrics of biodiversity impact). Gray ribbons are 90% credible intervals for the position of the line of best fit (i.e. mean; black line) across posterior iterations.

Supplementary Figure 6

a) Detection-corrected data, Sorensen dissimilarity

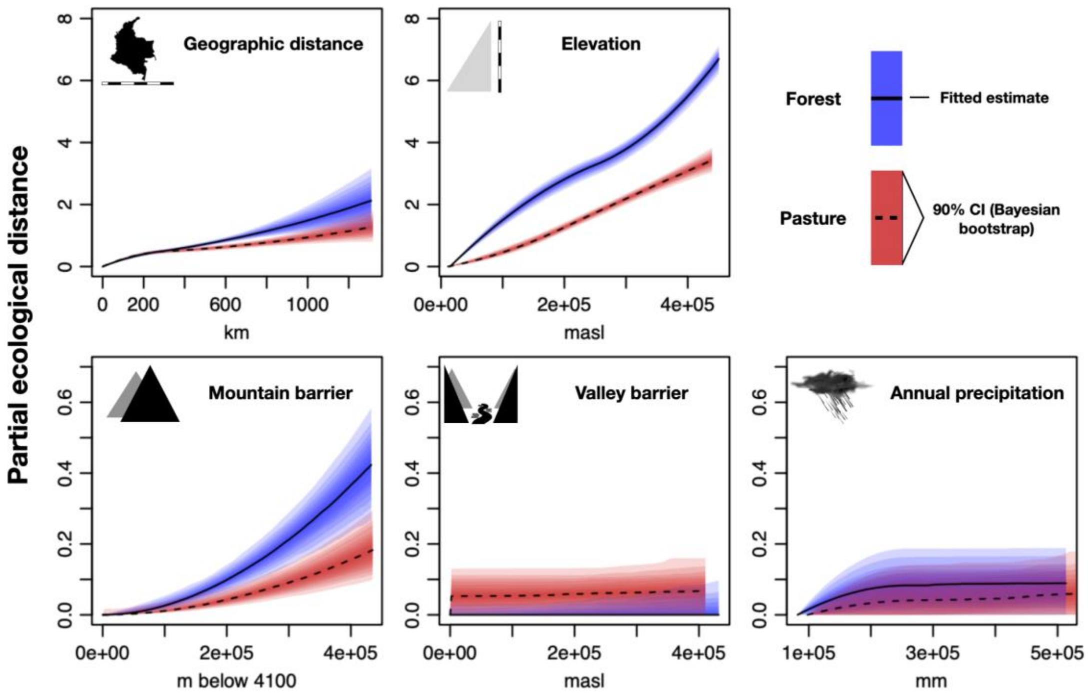

b) Raw data, Simpson dissimilarity

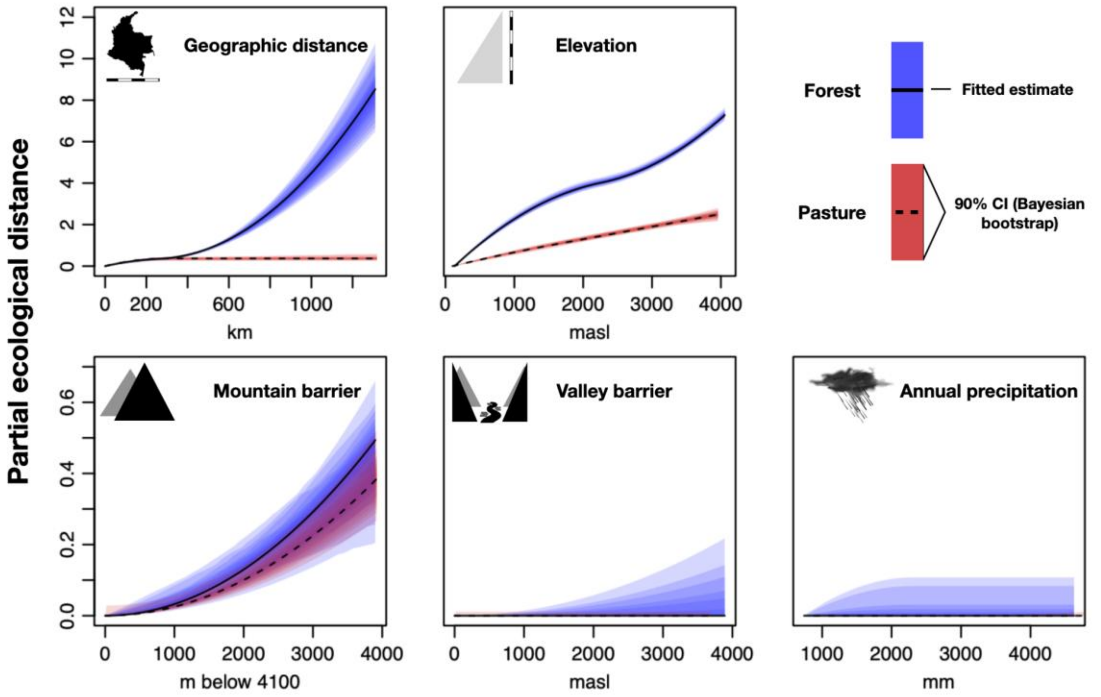

c) Detection-corrected data, Simpson dissimilarity

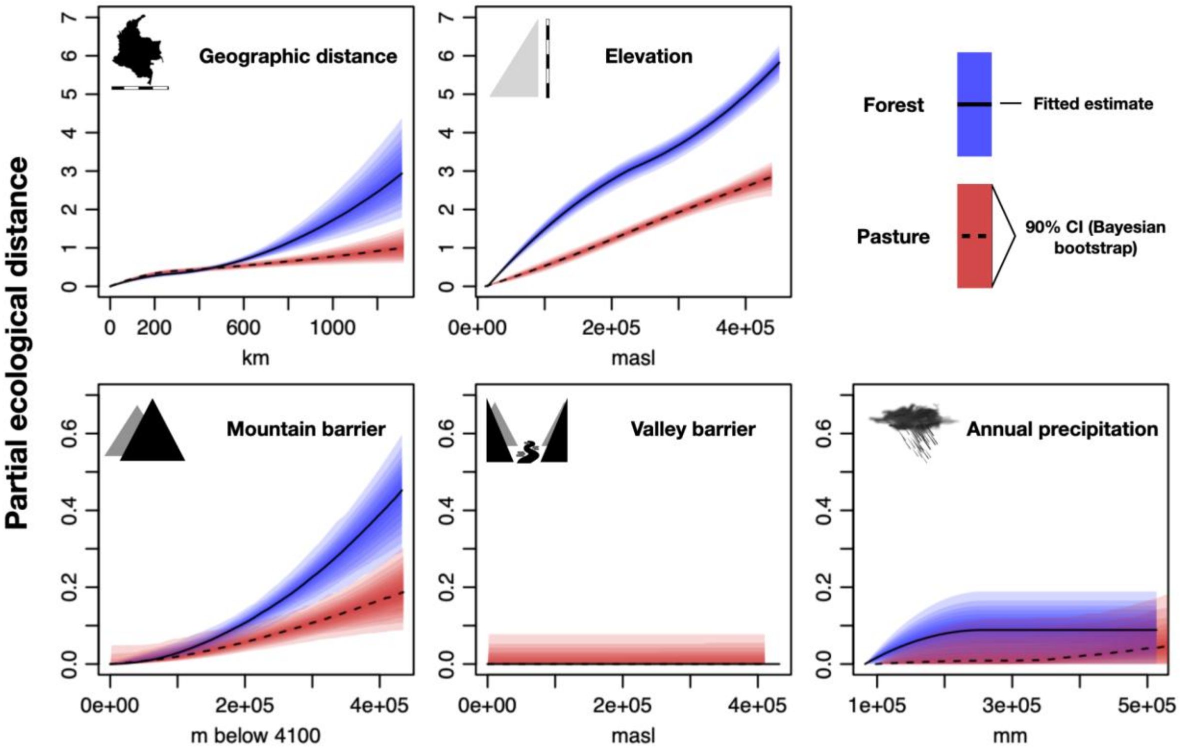

Supplementary Figure 6 Partial effects from Generalised Dissimilarity Models (GDMs) of (a) Sorensen dissimilarities based on detection-corrected data, (b) Simpson dissimilarities based on raw data, and (c) Simpson dissimilarities based on detection-corrected data. Main text figure 4 presents results for Sorensen dissimilarities based on raw data. Panel b indicates how the partial ecological distances vary along each respective gradient, based on a GDM fitted to the observed species assemblages at 848 cattle and pasture sampling points comprising 3357 point visits. Line of best fit (mean, black line) with 90% Credible Interval are shown.

Supplementary Table 1: Occupancy model parameters, priors, and posterior margins

				Posterior 90% CI	
**Model component**	**Parameter class**	**Parameter**	**Prior**	**.05 quantile**	**.95 quantile**
Occupancy	intercept	intercept	normal(-7, 2.5)	-9.57	-7.36
	slope	point is pasture	normal(0,1)	-1.23	0.19
		relative elevation	normal(0,5)	-1.08	-0.46
		relative elevation squared	normal(0,5)	-6.27	-5.42
		species reaches lowlands	normal(0,1)	-0.76	-0.06
		[relative elevation] x [species reaches lowlands]	normal(0,5)	-2.02	-1.48
		[relative elevation squared] x [species reaches lowlands]	normal(0,5)	0.62	1.22
		species elevational breadth	normal(0,1)	0.40	0.86
		[species elevational breadth] x [point is pasture]	normal(0,1)	0.42	0.78
		species elevational median	normal(0,1)	-0.19	0.42
		[species elevational median] x [point is pasture]	normal(0,1)	-0.56	-0.18
		species is migratory	normal(0,1)	-2.12	-1.05
		[species is migratory] x [point is pasture]	normal(0,1)	-0.33	0.34
		species body mass	normal(0,0.5)	-0.75	-0.06

		[species body mass] x [point is pasture]	normal(0,0.5)	-0.33	0.09
		species occurs in arid biomes	normal(0,1)	-0.48	0.39
		[species occurs in arid biomes] x [point is pasture]	normal(0,1)	-0.14	0.43
		species occurs in dry forest biome	normal(0,1)	0.27	0.75
		[species occurs in dry forest biome] x [point is pasture]	normal(0,1)	0.06	0.41
		species occurs in forest biomes	normal(0,1)	0.61	1.29
		[species occurs in forest biomes] x [point is pasture]	normal(0,1)	-0.70	-0.21
		[species occurs in forest biomes] x [species elevational median]	normal(0,1)	-0.37	0.15
		[species occurs in forest biomes] x [species elevational median] x [point is pasture]	normal(0,1)	0.08	0.47
		species is restricted to forest biomes	normal(0,1)	-0.30	0.32
		[species is restricted to forest biomes] x [point is pasture]	normal(0,1)	-1.21	-0.81
		[species is restricted to forest biomes] x [species elevational median]	normal(0,1)	0.11	0.57
		[species is restricted to forest biomes] x [species elevational median] x [point is pasture]	normal(0,1)	-0.25	0.06
		species is restricted to dry forest biomes	normal(0,1)	-0.67	0.21

		and/or is a floodplain specialist in Amazonia			
		[species is restricted to dry forest biomes and/or is a floodplain specialist in Amazonia] x [point is pasture]	normal(0,1)	0.13	0.79
		species is restricted to terra firme in Amazonia	normal(0,1)	-0.43	-0.01
		[species is restricted to terra firme in Amazonia] x [point is pasture]	normal(0,1)	-0.32	-0.01
		species is limited by a mountain barrier	normal(0,1)	-0.18	0.29
		[species is limited by a mountain barrier] x [point is pasture]	normal(0,1)	-0.41	-0.05
		species is limited by a valley barrier	normal(0,1)	-0.36	0.25
		[species is limited by a valley barrier] x [point is pasture]	normal(0,1)	-0.51	-0.12
		species is a carnivore	normal(0,0.5)	-0.94	0.11
		[species is a carnivore] x [point is pasture]	normal(0,0.5)	-0.65	0.05
		species is a frugivore or nectivore	normal(0,0.5)	0.22	0.79
		[species is a frugivore or nectivore] x [point is pasture]	normal(0,0.5)	-0.13	0.28
		species is a granivore	normal(0,0.5)	-0.65	0.14
		[species is a granivore] x [point is	normal(0,0.5)	-0.08	0.43

		pasture]			
		species is an insectivore	normal(0,0.5)	-0.55	-0.02
		[species is an insectivore] x [point is pasture]	normal(0,0.5)	-0.33	0.03
	monotonic effect[1]	distance from range	flat	-0.88	-0.66
	random effect standard deviations	intercept: by species	half-normal(0,2)	2.32	2.82
		intercept: by family	half-normal(0,2)	1.24	2.28
		intercept: by species-cluster	half-normal(0,3)	2.07	2.29
		intercept: by species-subregion	half-normal(0,3)	2.79	3.08
		slope, point is pasture: by species	half-normal(0,1)	1.56	1.92
		slope, point is pasture: by family	half-normal(0,1)	0.43	1.09
		slope, relative elevation: by species	half-normal(0,2)	2.91	3.90
		slope, relative elevation squared: by species	half-normal(0,2)	2.53	3.12
	random effect covariances	all	LKJ(1)		
Detection	intercept	intercept	normal(-3,1)	-3.63	-2.71
	slope	point is pasture	normal(0,0.75)	-0.04	0.34
		species body mass	normal(0,0.5)	-0.30	-0.01
		species elevational median	normal(0,0.5)	0.03	0.19
		species is migratory	normal(0,1)	-1.05	-0.52

		species is a carnivore	normal(0,0.5)	-0.46	0.15
		count time of day	normal(0,0.5)	-0.30	-0.24
		[count time of day] x [point elevation]	normal(0,0.5)	-0.02	0.02
		observer is SM	normal(0,0.25)	-0.05	0.08
		observer is JG	normal(0,0.25)	0.17	0.29
		observer is DPE	normal(0,0.25)	0.09	0.21
	random effect standard deviations	intercept: by species	half-normal(0,2)	0.66	0.89
		intercept: by family	half-normal(0,2)	0.49	0.99
		intercept: by [species x observer]	student_t(3, 0, 2.5)	0.65	0.77
		slope, point is pasture: by species	half-normal(0,1)	0.66	0.83
		slope, point is pasture: by family	half-normal(0,1)	0.19	0.57
		slope, count time: by species	half-normal(0,1)	0.20	0.25
	random effect covariances	all	LKJ(1)		

[1]: We used 12 bins for our monotonic effect, so an average estimate effect size of -0.76 between adjacent bins indicates a total difference of (12 - 1) * -0.76 = -8.36 between a species' core range and the outermost distance bin at 160 km outside the mapped range.

Appendix 1: Manual range additions

Against our buffered range maps (based on the range maps of Ayerbe-Quiñones[67]), our field data exposed errors of omission in 12 species, 5 of which we detected during a single expedition to the isolated Tamá massif on the Venezuelan border (3 of these were previously known from immediately adjacent Venezuela). We manually added range as follows, and we then buffered and clipped around our manual range additions according to the biogeographic buffering procedure described in the methods (i.e. clipping at a 160 km buffer beyond these range additions).

Birds of the Tamá Massif: We detected five species – *Aulacorhynchus calorhynchus*, *Grallaricula ferrugineipectus*, *Thripadectes virgaticeps*, *Coeligena bonapartei*, and *Thamnophilus unicolor* – on the Tamá massif, which straddles the Venezuelan border[105]. Ayerbe-Quiñones does not map them from this area, but three of them are known to occur in adjacent Venezuela. For each species, we manually added a polygon on the Tamá massif.

Crypturellus brevirostris: We detected this species on the southeastern flank of the Chiribiquete massif (Caquetá), > 400 km from all previous records[106]. We manually added a polygon along the southeastern flank of the Chiribiquete massif.

Entomodestes coracinus: We detected this species on the east slope of the West Andes in a region where Ayerbe-Quiñones maps this species as being (barely) restricted to the west slope. Our records coincide with numerous additional east-slope records, and we manually added polygons around known clusters of east-slope records[28].

Grallaricula flavirostris: We detected this species on the Serranía de Yariguíes, which is omitted from the Ayerbe-Quiñones map despite multiple specimen records[107]. We manually added a polygon on the Serranía de Yariguíes.

Hemitriccus striaticollis: This species is spreading in the Caquetá deforestation front, and our fieldwork produced the first records from the vicinity of Puerto Leguízamo[108]. We manually added a polygon encompassing the deforested pastures around Puerto Leguízamo.

Myrmotherula ambigua: We detected this species at Puerto Leguízamo, where it has been recorded previously[108]. We manually added a polygon extending westward from the range in Ayerbe-Quiñones to encompass recent records near Puerto Leguízamo, Puerto Asis, and Mocoa.

Sciaphylax castanea: We detected this species at Puerto Leguízamo, where it has been recorded previously[108]. We manually added a polygon extending southeastward from the range in Ayerbe-Quiñones to Puerto Leguízamo.

Thlypopsis superciliaris: We recorded this species at multiple locations in the western Andes. Ayerbe-Quiñones omits this species from the northern part of the West Andes in Colombia, despite its well documented occurrence in the area. We manually added a polygon around a cluster of eBird records in the departments of Antioquia, Risaralda, Caldas, and Chocó[28].

Appendix 2: Model development

Our occupancy model went through several rounds of revision before we arrived at the final model formulation. To promote transparent science and expose degrees of freedom in our approach that would otherwise remain hidden[96], we document our model-building process here. We note that the size and scope of our data collection effort was not strictly predetermined in advance; of necessity it was responsive to logistical conditions that we encountered in the field and was eventually curtailed by the COVID-19 pandemic. However, we confirm that we did not begin analyzing the data in earnest until the data collection effort was complete, and thus we avoided the risk that our pursuit of interesting results might inform our decision about when to stop data collection.

We initially fit a model (model 1) that differed from the final model presented in the paper as follows:

- It lacked a distance-to-range covariate for occupancy (but still used biogeographic clipping[28] at 160 km).
- It did not include occupancy covariates for limitation by generic mountain or valley barriers (or their interactions with pasture), and instead included separate terms for species that were a) restricted to the east side of the East Andes, b) restricted to the west side of the East Andes, c) restricted to the Santa Marta, d) absent from the West Andes, and e) absent from the East Andes, as well as their interactions with pasture.
- It lacked an effect of subregion-by-species on occupancy.

- It included a covariate indicating whether Amazonian species are specialists on floodplains, in addition to the covariate for species that are floodplain specialists, dry forest specialists, or both

We modified model 1 to yield model 2 as follows:

- We included a distance-to-range covariate with an associated slope that varied as a random effect of species. We made this decision after plotting a map of the point-scale community-averaged sensitivity (from model 1) across space. We noticed substantial artifacts on the map involving discontinuities where large numbers of species with coincident range limits all reached the edge of their 160-km range buffer. For example, one such discontinuity swept across the northern llanos, 160 km north of the mapped northern limit of a large number of highly sensitive Amazonian species.
- We replaced the several topographic barrier terms of model 1 with single terms for mountain-limited and valley-limited species. We made this decision after observing that the interaction between restriction to the Santa Marta (i.e. Santa Marta endemic species) and occurrence in pasture was very strongly negative, reflecting almost complete separation (i.e. nearly all of our records of Santa Marta endemic birds came from forest points). This separation is almost certainly a consequence of our limited number of total sampling points on the Santa Marta, as we sometimes detected Santa Marta endemic species in pastures incidentally. We further observed that the slopes estimated for the remainder of

the original topographic barrier terms (and their interactions with pasture) were not clearly different.

This model ran without divergences but experienced moderate convergence problems, particularly in the standard deviation of the random effect of distance-to-range by species (r-hat 1.2).

We modified model 2 to yield model 3, our final model upon which we base our inference, as follows:

- We removed the random slope by species on the distance-to-range effect and replaced it with a flexible monotonic effect that was fully pooled across species. To include a flexible effect with a monotonicity constraint in the brms modeling framework, we binned distance-to-range in 20-km bins and fitted a monotonic effect of this ordinal predictor[82].
- We included a random effect of subregion-by-species on occupancy. We made this decision after conducting posterior predictive checking for spatial autocorrelation based on join-count statistics. Posterior predictive checks revealed that our model underestimated the spatial autocorrelation in our data and that this underestimation was most severe at spatial scales of roughly 20 km. Therefore, we defined subregions by grouping points at the 20 km scale, and we included a random effect on occupancy of subregion-by-species.

Appendix 3: posterior predictive checks

We assessed model adequacy with multiple posterior and mixed predictive checks[97] tailored to detect specific forms of misspecification. Because our model is fit to over two million data points, we have tremendous power to detect mild forms of misspecification. Therefore, our focus is not on ensuring that we "pass" the checks, but rather on guiding model development to arrive at an adequate model without obvious avenues for further improvement. Here, we describe the checks and present their results alongside the descriptions.

Sum-of-Q

Does the model adequately predict the total number of species-point combinations at which we obtained at least one detection? By comparing the observed number of points with at least one detection to the posterior predictive distribution that our model yields for this quantity, we observe no indication of misspecification (Supplementary Figure 7).

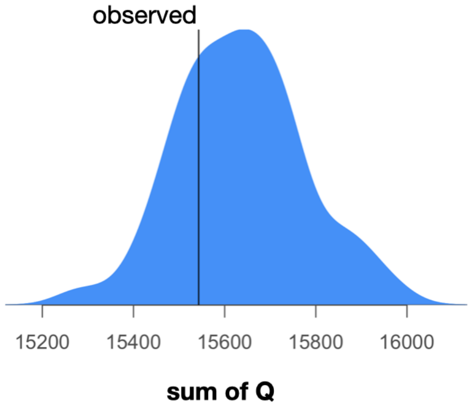

Supplementary Figure 7 The observed number of points with at least one detection (vertical line) plotted alongside its posterior predictive distribution (blue density plot).

Detection-history cohorts

Does the model adequately predict the total counts of each of the 16 possible 4-visit detection histories? This check, originally proposed by Mackenzie & Bailey[109], is standard in the occupancy modeling literature. We find that our model moderately underpredicts the number of detection histories with four detections, and moderately overpredicts the number of detection histories with two detections (Supplementary Figure 8), suggestive of unmodeled heterogeneity in detection probabilities. Occupancy models are typically robust to moderate levels of detection heterogeneity[110], and no additional available covariates are obvious candidates to explain this residual variation.

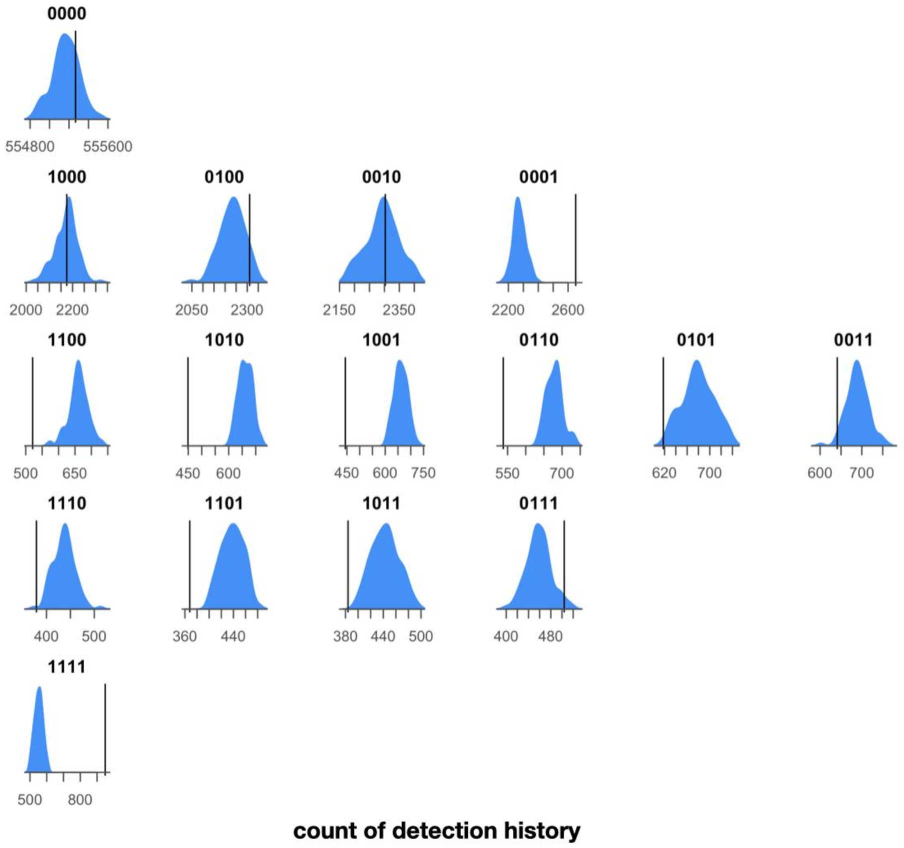

Supplementary Figure 8 The observed counts of each possible four-visit detection history (vertical lines) plotted alongside their posterior predictive distributions (blue density plots).

Spatial structure

Does the model adequately capture the spatial structure of our data? For each species, we compute join-count test statistics[111] based on detection/nondetection data using multiple binary weights matrices that define neighbors based on distance thresholds of 5 km, 10 km, 20 km, 50 km, or 100 km. We compute the join-count statistics at the cluster level because the model already contains a highly flexible random effect of species-by-cluster to handle within-cluster correlations. For each distance threshold, and for each species modeled as potentially occurring in the vicinity of at least three clusters, we assess the quantile of the posterior predictive distribution into which the observed joint-count statistic falls; such quantiles are sometimes referred to as *Bayesian p-values*[97]. We repeat this calculation forest clusters only, pasture clusters only, and all clusters combined. The distribution of Bayesian p-values across species for each distance threshold gives no indication that our model inadequately captures the spatial structure in the data (Supplementary Figure 9).

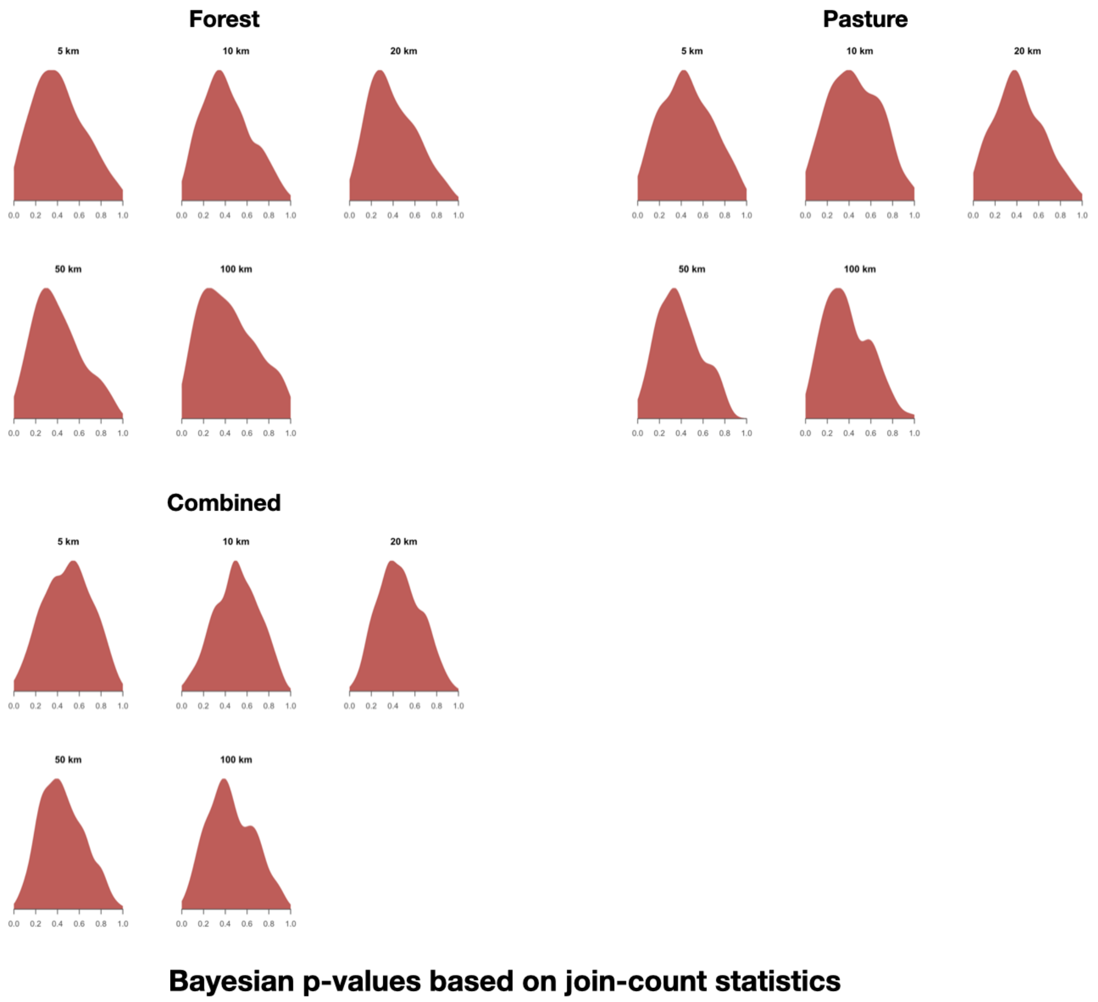

Supplementary Figure 9 Distributions across species of Bayesian p-values based on join-count statistics at five distance thresholds, shown for forest clusters, pasture clusters, and all clusters combined. Excesses of p-values near zero or one would indicate misspecification; here we have no evidence for that. The excess of p-values near 0.5 is expected in Bayesian posterior predictive checking and does not suggest anything pathological[91].

Phylogenetic signal

Does the model adequately capture phylogenetic signal implied by the data? For each of the seven random-effect terms grouped by species, we computed Pagel's lambda[112,113] for the species-specific parameters and compared to a reference

distribution. At each posterior iteration, we computed Pagel's lambda by pairing our posterior samples with a different equiprobable phylogenetic hypothesis from Pulido-Santacruz & Weir[114], which updates the Hackett-backbone[115] phylogenetic hypotheses of Jetz et al[116] with a better-resolved phylogeny of the Furnariidae[117]. To ensure that the check is sensitive, we repeated the process while progressively filtering to include only increasingly data-rich species (species detected on at least 0, 10, 50, or 100 points). Including too many data-poor species can cause the fitted distribution to be dominated by poorly informed values determined primarily by the hyperparameters, while including too few species can lead to excessive noise in the mixed predictive distribution for Pagel's lambda. Supplementary Figure 10 shows the distribution of Pagel's lambda for all seven of the species-level random effects. We then compared these values to values from a reference distribution generated by pairing the same phylogenetic hypotheses with hypothetical random effect values simulated directly from a Normal distribution, and we show the mixed predictive distribution for the difference between Pagel's lambda calculated from the fitted random effects and the re-simulated random effects in Supplementary Figure 11.

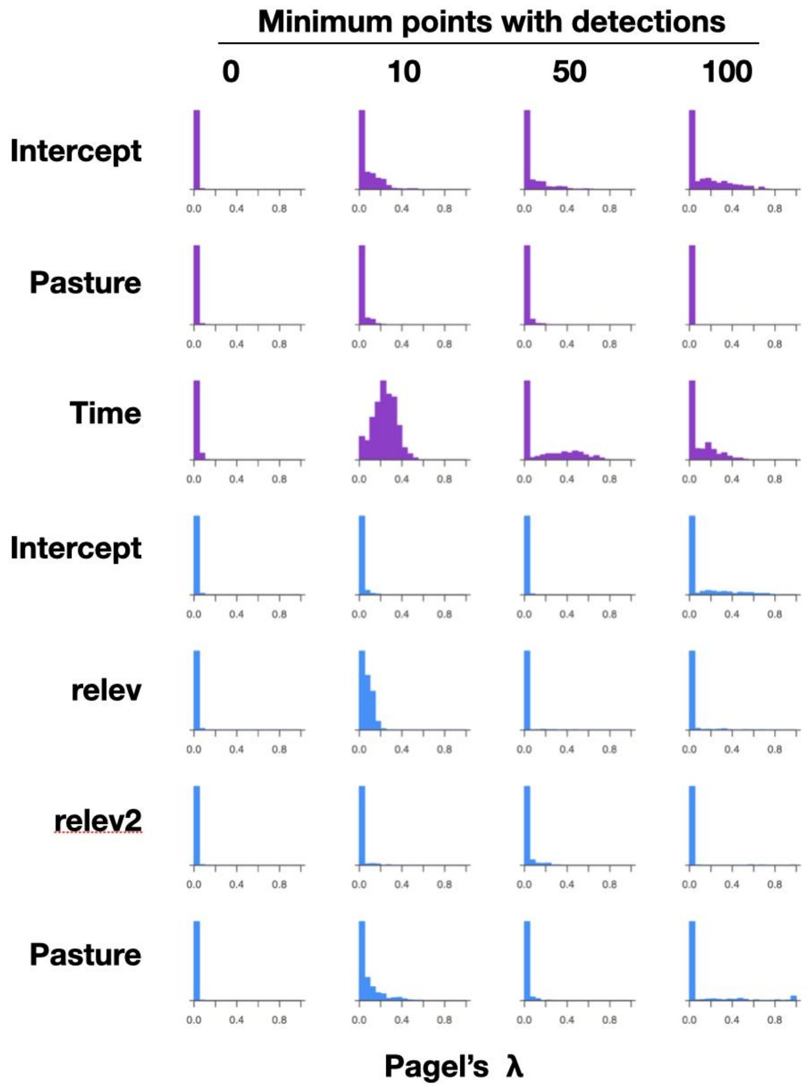

Supplementary Figure 10 Posterior distributions of Pagel's lambda over the fitted random effects. Columns show Pagel's lambda computed over species pools restricted to species detected on progressively larger numbers of total points. Rows show different random effect terms grouped by species, with effects on detection plotted in purple and effects on occupancy in blue. In order, these are the random effect of species on: the detection intercept, the detection effect of pasture versus forest, the detection effect of time-of-day, the occupancy intercept, the occupancy effect of relative elevation, the occupancy effect of relative elevation squared, and the occupancy effect of pasture versus forest. Importantly, we have no clear evidence of unmodeled phylogenetic signal in any of the random effect terms, as all random effects yield posteriors that include values near zero for Pagel's lambda.

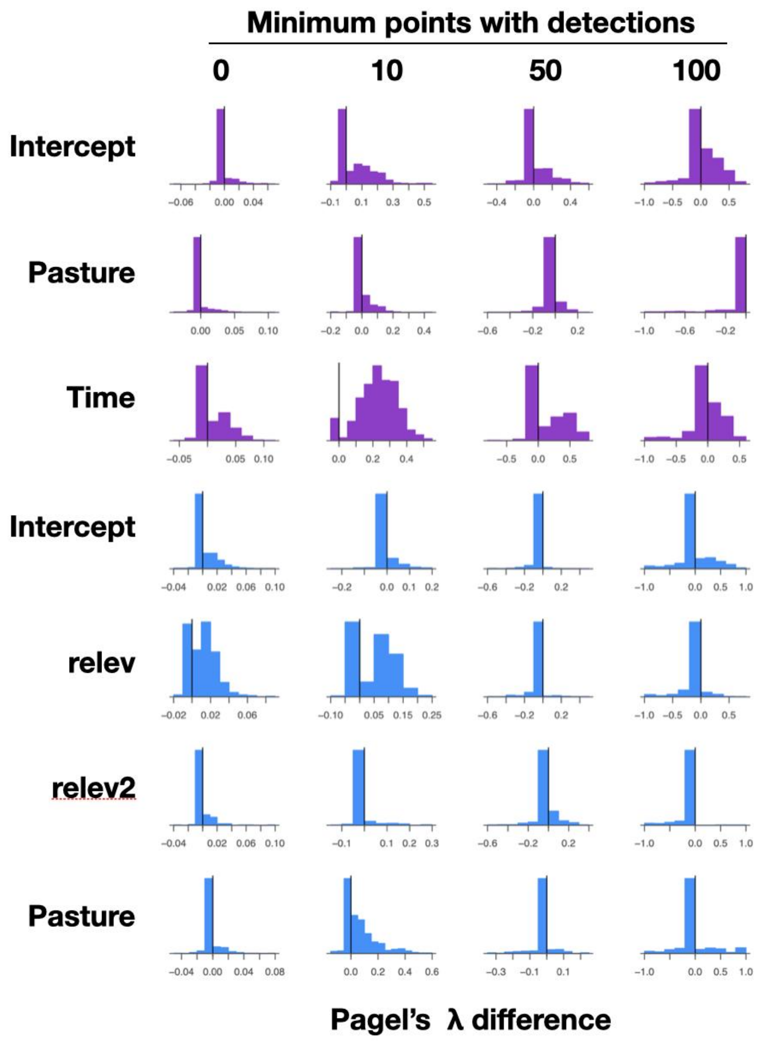

Supplementary Figure 11 Posterior distributions for the difference between Pagel's lambda over the fitted random effects and Pagel's lambda over random effects that are iid Guassian by construction. Rows and columns are as in Supplementary Figure 10.

Random effect normality

Do the model's Gaussian hyper-distributions adequately capture the shape of the group-level variation in our data? At each posterior iteration, we compute an Anderson-Darling test statistic[118] over the elements of each random effect vector in our model. This test statistic is used in frequentist applications to detect evidence of non-normality.

However, rather than convert the test statistic to a frequentist p-value, we treat the Anderson-Darling test statistic as a generated quantity to compare iteration-wise to Anderson-Darling test statistics computed from simulated vectors of the same length whose elements are independent and identically distributed Gaussian variates. Because the predictive distribution for the difference between test statistics computed on our fitted data and simulated Gaussian data consistently overlaps zero (Supplementary Figure 12), we find no evidence to suggest that our assumptions of normally distributed random effects are problematic. For the same reason discussed above under Pagel's lambda, for the seven random effects grouped by species, we also repeat this check while filtering to species detected on progressively larger numbers of points (Supplementary Figure 13), again finding no evidence to suggest that normally distributed random effects are problematic.

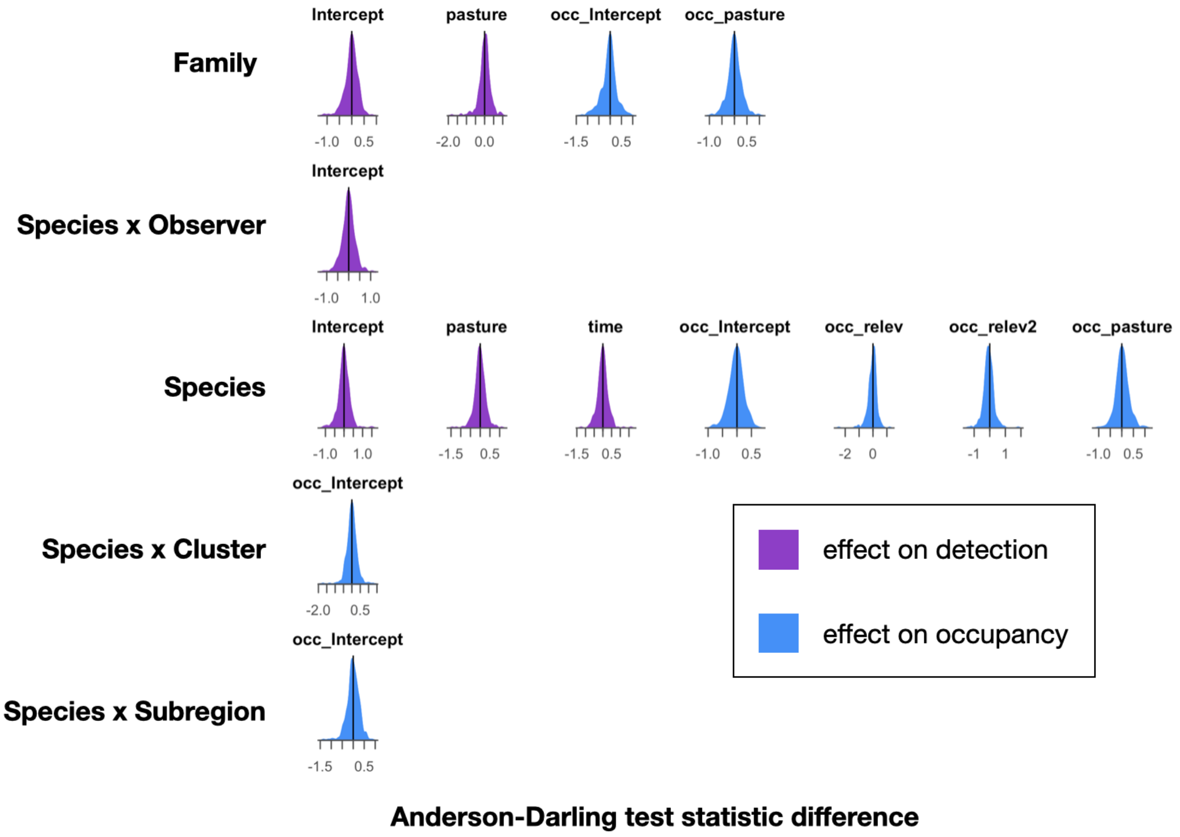

Supplementary Figure 12 Posterior distributions for the difference between Anderson-Darling test statistics computed over the fitted random effect vectors and over independent and identically distributed Gaussian vectors of the same length. Each row presents results for the random effects corresponding to a different grouping factor. Because all of these distributions cover zero (vertical black lines), there is no evidence that our assumption of normally distributed random effects is inadequate.

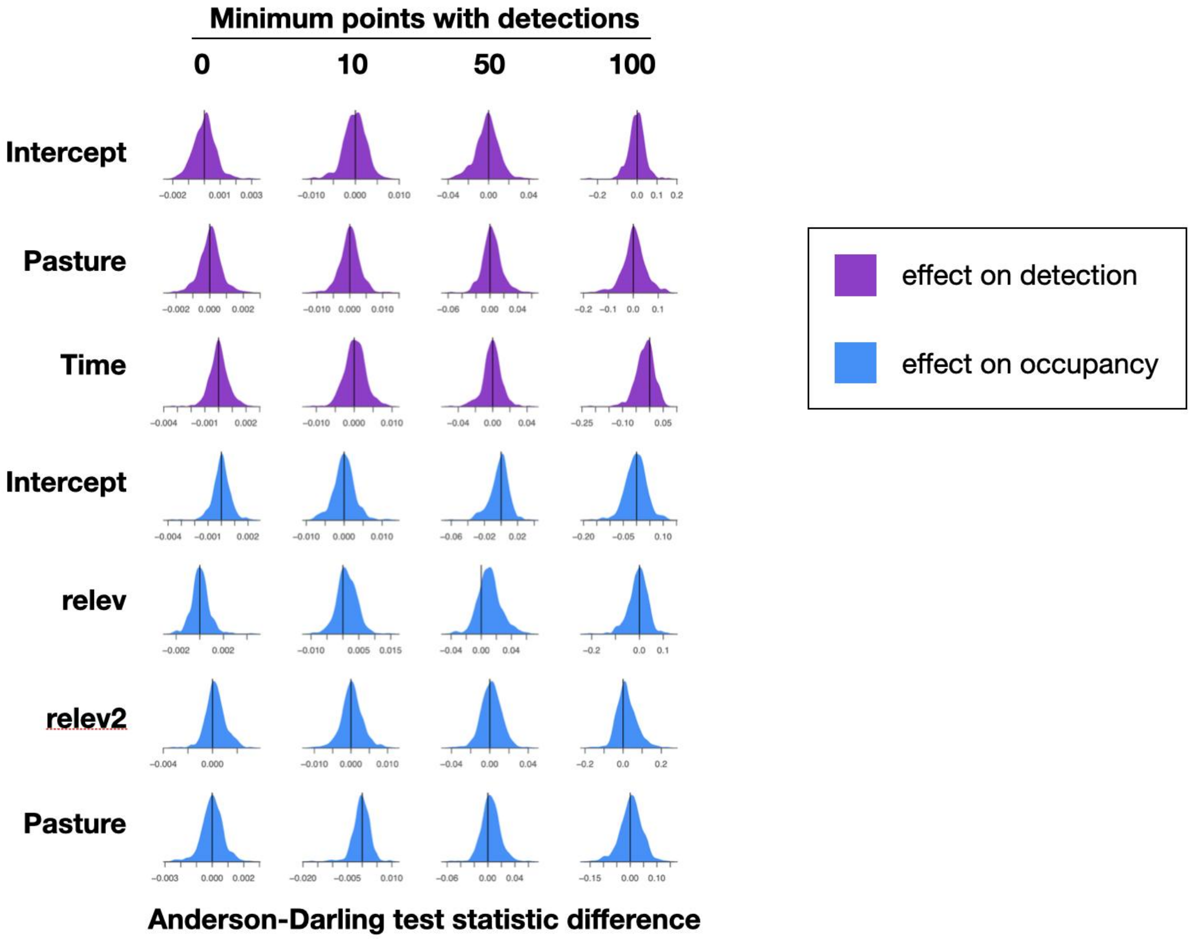

Supplementary Figure 13 Posterior distributions for the difference between Anderson-Darling test statistics computed over the fitted random effect vectors and simulated iid Gaussian vectors of the same length. Here, we show only random effects grouped by species, and we progressively filter to include only species with greater numbers of total detections. Note that the first column is equivalent to the third row of Supplementary Figure 12.

Supplementary References

105. Socolar, J. B. & Peña, A. Noteworthy bird records from the Tamá massif and adjacent areas, Norte de Santander, Colombia. *Ornitol. Colomb.* 17–25 (2022) doi:10.59517/oc.e542.

106. Socolar, J. B., Fernando-Castaño, J. & Arango, J. Noteworthy bird records from the Araracuara area, Amazonas and Caquetá, Colombia. *Ornitol. Colomb.* 2–10 (2022) doi:10.59517/oc.e538.

107. Donegan, T., Avendaño, J., Briceño Lara, E. & Huertas, B. Range extensions, taxonomic and ecological notes from Serranía de los Yariguíes, Colombia's new national park. *Bull. Br. Ornithol. Club* **127**, 172–213 (2007).

108. Acevedo-Charry, O. *et al.* Avifauna del interfluvio de la cuenca media Caquetá Putumayo (Japurá-Içá), al sur de la Amazonia colombiana y su respuesta a la huella humana. *Rev. Acad. Colomb. Cienc. Exactas Físicas Nat.* **45**, 229–249 (2021).

109. MacKenzie, D. I. & Bailey, L. L. Assessing the fit of site-occupancy models. *J. Agric. Biol. Environ. Stat.* **9**, 300–318 (2004).

110. Guillera-Arroita, G., Lahoz-Monfort, J. J., MacKenzie, D. I., Wintle, B. A. & McCarthy, M. A. Ignoring Imperfect Detection in Biological Surveys Is Dangerous: A Response to 'Fitting and Interpreting Occupancy Models'. *PLOS ONE* **9**, e99571 (2014).

111. Cliff, A. D. & Ord, J. K. *Spatial Processes: Models & Applications*. (Pion, 1981).

112. Pagel, M. Inferring the historical patterns of biological evolution. *Nature* **401**, 877–884 (1999).

113. Revell, L. J. phytools: an R package for phylogenetic comparative biology (and other things). *Methods Ecol. Evol.* **3**, 217–223 (2012).

114. Pulido-Santacruz, P. & Weir, J. T. Extinction as a driver of avian latitudinal diversity gradients. *Evolution* **70**, 860–872 (2016).

115. Hackett, S. J. *et al.* A Phylogenomic Study of Birds Reveals Their Evolutionary History. *Science* **320**, 1763–1768 (2008).

116. Jetz, W., Thomas, G. H., Joy, J. B., Hartmann, K. & Mooers, A. O. The global diversity of birds in space and time. *Nature* **491**, 444–448 (2012).

117. Derryberry, E. P. *et al.* Lineage Diversification and Morphological Evolution in a Large-Scale Continental Radiation: The Neotropical Ovenbirds and Woodcreepers (Aves: Furnariidae). *Evolution* **65**, 2973–2986 (2011).

118. Anderson, T. W. & Darling, D. A. Asymptotic Theory of Certain ‘Goodness of Fit’ Criteria Based on Stochastic Processes. *Ann. Math. Stat.* **23**, 193–212 (1952).
