# Supplementary material for: Tropical biodiversity loss from land-use change is severely underestimated by local-scale assessments
Source: Nat Ecol Evol. 2025 Jul 22;9(9):1643–55. doi: 10.1038/s41559-025-02779-4 (PMC12420384; doi:10.1038/s41559-025-02779-4)
Supplement: Supplementary file 2 — Reporting Summary [file 41559_2025_2779_MOESM2_ESM.pdf]

nature portfolio

Corresponding author(s): Edwards

Last updated by author(s): May 16, 2025

Reporting Summary

Nature Portfolio wishes to improve the reproducibility of the work that we publish. This form provides structure for consistency and transparency in reporting. For further information on Nature Portfolio policies, see our Editorial Policies and the Editorial Policy Checklist.

Statistics

For all statistical analyses, confirm that the following items are present in the figure legend, table legend, main text, or Methods section.

n/a	Confirmed	
☐	☒	The exact sample size (*n*) for each experimental group/condition, given as a discrete number and unit of measurement
☒	☐	A statement on whether measurements were taken from distinct samples or whether the same sample was measured repeatedly
☐	☒	The statistical test(s) used AND whether they are one- or two-sided<br>*Only common tests should be described solely by name; describe more complex techniques in the Methods section.*
☐	☒	A description of all covariates tested
☐	☒	A description of any assumptions or corrections, such as tests of normality and adjustment for multiple comparisons
☐	☒	A full description of the statistical parameters including central tendency (e.g. means) or other basic estimates (e.g. regression coefficient) AND variation (e.g. standard deviation) or associated estimates of uncertainty (e.g. confidence intervals)
☒	☐	For null hypothesis testing, the test statistic (e.g. F, t, r) with confidence intervals, effect sizes, degrees of freedom and P value noted<br>*Give P values as exact values whenever suitable.*
☐	☒	For Bayesian analysis, information on the choice of priors and Markov chain Monte Carlo settings
☐	☒	For hierarchical and complex designs, identification of the appropriate level for tests and full reporting of outcomes
☐	☒	Estimates of effect sizes (e.g. Cohen's *d*, Pearson's *r*), indicating how they were calculated

Our web collection on statistics for biologists contains articles on many of the points above.

Software and code

Policy information about availability of computer code

Data collection	N/A
Data analysis	R, Stan, brms

For manuscripts utilizing custom algorithms or software that are central to the research but not yet described in published literature, software must be made available to editors and reviewers. We strongly encourage code deposition in a community repository (e.g. GitHub). See the Nature Portfolio guidelines for submitting code & software for further information.

Data

Policy information about availability of data

All manuscripts must include a data availability statement. This statement should provide the following information, where applicable:

- Accession codes, unique identifiers, or web links for publicly available datasets
- A description of any restrictions on data availability
- For clinical datasets or third party data, please ensure that the statement adheres to our policy

All data will be archived on Figshare and made available at publication. The codebase for cleaning and handling the data are all made available at https://github.com/jsocolar/colombiaBeta

Research involving human participants, their data, or biological material

Policy information about studies with human participants or human data. See also policy information about sex, gender (identity/presentation), and sexual orientation and race, ethnicity and racism.

Reporting on sex and gender	N/A
Reporting on race, ethnicity, or other socially relevant groupings	N/A
Population characteristics	N/A
Recruitment	N/A
Ethics oversight	N/A

Note that full information on the approval of the study protocol must also be provided in the manuscript.

Field-specific reporting

Please select the one below that is the best fit for your research. If you are not sure, read the appropriate sections before making your selection.

☐ Life sciences ☐ Behavioural & social sciences ☒ Ecological, evolutionary & environmental sciences

For a reference copy of the document with all sections, see nature.com/documents/nr-reporting-summary-flat.pdf

Ecological, evolutionary & environmental sciences study design

All studies must disclose on these points even when the disclosure is negative.

Study description	Fieldwork was carried out across the majority of Colombia's major biogeographic regions, namely the three Andean Cordilleras, the Santa Marta Massif, and the lowlands of the Amazon, Llanos, and Magdalena valley. At each study site, survey points were established in natural forest (or paramo at high elevations), and we endeavoured to pair any forest point with a pasture point at the same elevation within a site. Points were installed in clusters of three sampling points with at least 200 metres separating each point (we account for the clustered design in our statistical analysis), and a minimum of 500 m separating clusters.
Research sample	We sampled 848 points, using a repeated point count design, with each point sampled four times across consecutive days. Based on published range maps and habitat, we sampled in range of 1614 species of bird, with 971 species detected on at least one point count. In total 3357 point-visits were completed, representing 560 hours of observation.
Sampling strategy	Broadly, the sampling strategy aimed to capture as much biogeographic variation as possible, by sampling as many distinct biogeographies as was logistically feasible. The central comparison in the manuscript is the change in the community composition between forest and pasture, and we therefore endeavoured to pair each pasture point at a site with a corresponding forest point at a similar elevation (to provide a like-for-like comparison between pasture and forest communities). The exception to this rule occurred at one Amazonian site where eight forest clusters were established, but there was only enough nearby pasture to establish three pasture clusters. Five additional pasture clusters were established at similar elevations, but at a separate site 320 km to the north.
Data collection	Data were collected by five expert observers (JBS, SCM, DPE, JJG, OG) in repeated point counts carried out on consecutive or near-consecutive days. Each point count consisted for 10-minute 100 m radius counts, carried out from the onset of the dawn chorus to afternoon. At some of the highest sites in the paramo, where bird activity does not fall off sharply during the afternoon, we conducted point counts into mid-afternoon. At a small minority of points points were only visited two or three times due to logistical challenges (illness, guerilla activity, etc.). During point counts, sound was recorded continuously to allow identification of any unknown sounds afterwards with reference to sound libraries (e.g. Xeno Canto) and in consultation with experts.
Timing and spatial scale	For any individual point, all counts were carried out on consecutive or near-consecutive days. Data are analysed using detection-occupancy models, and a central assumption made by this class of model is that of closure over the sampling period, which we meet by sampling in this timeframe. We sampled during the morning to coincide with bird activity, and accomodate temporal variation in detection over the course of the morning in our statistical model.
Data exclusions	There were no data exclusions.
Reproducibility	All data required for analysis will be archived on Figshare and made freely available on acceptance for publication. The codebase for carrying out all analyses is archived at: https://github.com/jsocolar/colombiaBeta
Randomization	Within a site we endeavoured to have paired forest-pasture samples at similar elevations. Point visits were randomised each day such that each point recieved a mix of both early and late visits. We have a statistical model that includes a large number of terms relating to potential confounding factors (most obviously habitat, but also time of day, observer) and site and cluster-specific random effects.

Blinding	It would not be possible to blind observers in a field study like this.

Did the study involve field work? ☒ Yes ☐ No

Field work, collection and transport

Field conditions	Fieldwork was carried out across the major biogeographic regions of Colombia, and comprised a broad range of bioclimatic conditions from the tropical lowlands of the Amazon and Magdalena through to sub-montane and montane regions in the three Andean Cordilleras.
Location	Colombia
Access & import/export	There was no import/export of samples. Where we worked within the limits of Parques Nacionales Naturales de Colombia, this was done with permission of the park authorities.
Disturbance	There was limited disturbance caused by the study. Trails were cut to access points in the interior of forest, but the impacts of this are short-lived.

Reporting for specific materials, systems and methods

We require information from authors about some types of materials, experimental systems and methods used in many studies. Here, indicate whether each material, system or method listed is relevant to your study. If you are not sure if a list item applies to your research, read the appropriate section before selecting a response.

Materials & experimental systems

n/a	Involved in the study	
☒	☐	Antibodies
☒	☐	Eukaryotic cell lines
☒	☐	Palaeontology and archaeology
☐	☒	Animals and other organisms
☒	☐	Clinical data
☒	☐	Dual use research of concern
☒	☐	Plants

Methods

n/a	Involved in the study	
☒	☐	ChIP-seq
☒	☐	Flow cytometry
☒	☐	MRI-based neuroimaging

Animals and other research organisms

Policy information about studies involving animals; ARRIVE guidelines recommended for reporting animal research, and Sex and Gender in Research

Laboratory animals	N/A
Wild animals	No animals were captured in the course of the study. Data consist purely of observations and field sound recordings.
Reporting on sex	N/A
Field-collected samples	N/A
Ethics oversight	N/A

Note that full information on the approval of the study protocol must also be provided in the manuscript.

Plants

Seed stocks	N/A
Novel plant genotypes	N/A
Authentication	N/A
